# Deterministic Foreground Calibration of Gain and Integral Nonlinearity in Constant-Slope Digital-to-Time Converters for Fractional Output Dividers

**DOI:** 10.3390/s26196091

**Published:** 2026-09-25

**Authors:** Saeid Karimpour, Emmanuel Nti Darko, Degang J. Chen

**Affiliations:** Department of Electrical and Computer Engineering, Iowa State University, Ames, IA 50011, USA; ntidarko@iastate.edu (E.N.D.); djchen@iastate.edu (D.J.C.)

**Keywords:** on-chip self-calibration, time interval measurement, digital-to-time converter, fractional output divider, bit-weight mismatch extraction, successive approximation register, femtosecond resolution, sensor clocking, clock generation, aperture jitter

## Abstract

Fractional output dividers (FODs) derive many clock domains from one integer-N phase-locked loop, but the gain error and integral nonlinearity (INL) of their digital-to-time converter (DTC) limit spectral purity. Existing FOD calibrations estimate the INL statistically, with no deterministic step count or residual bound. This paper presents a deterministic foreground calibration that measures the DTC bit weights in the delay domain. Reconfiguring an R-2R ladder bit by bit inside a constant-slope DTC maps each weight to a SAR-driven edge comparison against a replica chain, so the five mismatch-dominant weights are extracted inside a fixed 124-decision sequence and corrected through a 32-entry look-up table. Allocating one input period to discharge and N−1 periods to DAC settling enables a 10 GHz input. Exact conditions are derived for gain measurement, offset–gain convergence, and weight extraction, together with the smooth curvature that per-bit calibration cannot observe. Transistor-level simulations in 22 nm FDSOI show chain tracking of 0.94 and offset–gain convergence below half the 12.2 fs calibration step. A 200-die system-level Monte Carlo anchored to these statistics reduces the median peak INL from 4.1 to 0.32 LSB (24.4 fs LSB) and the worst fractional spur from −75.5 dBc to the −91 dBc quantization floor after 0.59 ms of calibration; the resulting 28 fs rms edge jitter permits 95 dB aperture-limited SNDR in a sampled sensor front end at 100 MHz.

## 1. Introduction

Modern systems-on-chip (SoCs) integrate processors, data converters, memory interfaces, radio front ends, and—increasingly—a heterogeneous set of sensor channels (imaging, ranging, inertial, biopotential) that each require an independently programmable clock. Duplicating a fractional-N phase-locked loop (PLL) per domain is costly in area and power, and closely spaced LC oscillators suffer injection pulling. A widely adopted alternative is to distribute one high-quality integer-N PLL clock and to synthesize each domain frequency with an open-loop fractional output divider (FOD): a multi-modulus divider (MMD) controlled by a delta-sigma modulator (DSM), followed by a digital-to-time converter (DTC) that cancels the instantaneous quantization error of the division [1,2]. The FOD switches frequency in nanoseconds because no loop must resettle, which makes it attractive for dynamic voltage and frequency scaling.

The DTC is the accuracy bottleneck of every FOD. Its gain error converts directly into deterministic jitter, and its integral nonlinearity (INL) generates fractional spurs that routinely dominate the output spectrum [1,2,3]. Because the FOD is open loop, the classical PLL feedback path that reveals DTC gain mismatch is unavailable, and a dedicated error detection mechanism must be constructed [4].

### 1.1. Relevance to Sensor Systems

Clock generation is not peripheral to sensing. In one large class of sensors the measurand is itself encoded in time, and in almost every other class the sampling clock sets the achievable noise floor. Both dependencies are quantitative, and both are governed by exactly the quantities this paper measures and calibrates.

The first dependency is direct. Time-of-flight sensing—optical and ultrasonic ranging, and pulsed time-domain readout generally—maps an interval Δt onto a distance through Δr=cΔt/2 [5], and pulsed readouts rely on programmable temporal gating, enabling the detector only within a narrow window placed around the expected echo [5]. A DTC is the natural generator of such programmed delays, and there its placement accuracy—the integral nonlinearity calibrated in this paper—is what matters. Its step size is not a sensor resolution. The single-shot precision of a pulsed-LiDAR channel combines the laser pulse width, the detector jitter, and the time converter error in quadrature and is usually dominated by the pulse width, and the time converter INL enters that budget as an explicit term that is conventionally corrected after the fact, by code density measurement of the bin widths or by dithering [5]. The contribution of this work to such a system is correspondingly specific: a delay generator whose nonlinearity is measured and corrected on chip, deterministically, with no reference other than the input clock. The comparison used for that purpose resolves the difference between two internal edges only within the ±1.56 ps span of the calibration vernier; it is not a general time interval meter.

The second dependency is indirect but far more widespread. Any sensor whose analog front end is sampled inherits the timing uncertainty of its sampling clock as an aperture error. For a sinusoidal input at frequency $f_{\mathrm{in}}$ sampled with RMS timing uncertainty $\sigma_{t}$, the aperture-limited signal-to-noise ratio is [6,7]

(1) $${\mathrm{SNR}}_{\mathrm{ap}}\;=\;-20\log_{10}\;\left(2\pi f_{\mathrm{in}}\;\sigma_{t}\right),$$

which is independent of the converter’s own resolution and therefore decides whether a high-resolution sensor ADC delivers its nominal bits. The relation depends on the jitter only through its total power, so it is insensitive to how that power is distributed in frequency [7]; for deterministic, INL-induced jitter, the same power appears as spurs, and the relation then bounds the signal-to-noise-and-distortion ratio (SNDR). The finding that this term is the operative limit is an empirical finding as much as an analytic one: across a survey of more than 150 reported converters, the achieved resolution falls by approximately one bit for each doubling of the sampling rate, a slope attributed to aperture uncertainty, with the best converters clustered at 0.5–2 ps of aperture jitter [8]. For the remainder of this paper, the relevant $\sigma_{t}$ is the total edge jitter that the DTC adds: the random floor in Section 3.4 (26.8 fs with the supply term at its one-LSB budget) plus the deterministic residue of the INL. The system-level evaluation in Section 5.7 puts the latter at 7.4 fs rms after calibration, against 53 fs with gain calibration alone, so the DTC contribution falls from 60 to 28 fs rms. By (1) this raises the aperture-limited SNDR the divider permits from 88.5 to 95.2 dB at a 100 MHz input, and from 68.5 to 75.2 dB at 1 GHz. These figures bound the divider’s own contribution; the jitter of the reference clock and of any buffering adds to them. The same relation turns the supply noise budget in Section 3.4 into a sensor system requirement: at the simulated 0.25 ps per mV supply sensitivity, one millivolt of unregulated supply noise adds 250 fs rms and would cap the aperture-limited SNR at 56 dB for a 1 GHz input.

The third connection is architectural. A sensor fusion SoC is the canonical instance of the many independent clock domains problem that motivates the FOD in the first place: imaging, ranging, inertial, and acoustic channels run at unrelated sample rates, are reprogrammed as operating modes change, and—where several channels form an array whose outputs are combined coherently—must hold a defined phase relationship to one another. The FOD supplies per-domain frequency agility in nanoseconds without a per-domain PLL, and the drift analysis in Section 6 shows why calibration matters for the array case specifically: drift common to every output edge is a static phase offset rather than jitter, so it perturbs only inter-channel skew, whereas the per-code bit-weight error removed by the calibration reported here appears directly in each channel’s own timing. Finally, the calibration is itself a consumer of sensors: the refresh of the offset and gain codes that temperature drift requires (Section 6) is scheduled from an on-chip temperature reading, so the timing reference and the sensor that keeps it calibrated close a single loop.

### 1.2. Related Work

Reported FOD calibrations have evolved along a clear trajectory. Replica-DTC schemes detect the gain error by comparison with a complementary copy of the DTC, at the cost of area, power, and replica mismatch [9]. Replica-free gain calibration removes the copy by comparing the main DTC at code zero and full code, but the comparison is only exercised on delta-sigma overflow events, which slows convergence for unfavorable frequency words, and the INL is not addressed; reported spur floors are near −65 dBc [3]. Auxiliary-PLL techniques add a frequency-domain filter that tracks the carrier and exposes the spurs, enabling background zeroth/first/second-order polynomial INL correction and pushing worst-case spurs below −80 dBc, at the cost of an extra PLL and per-segment look-up tables [2,4]. Most recently, split-DTC architectures divide the DTC into two halves that reference each other through a bang-bang phase detector, with redundant decoding and pseudo-random selection decorrelating the internal states so that sign least mean square (sign-LMS) engines can estimate gain and INL in the background [1,10]; a foreground variant applies the same mutual comparison and sign-LMS principle to two main DTCs [11].

Common to all is that the INL is estimated statistically: an LMS loop converges under a decorrelation assumption, its convergence time depends on the input statistics, and no closed form bounds the residual. Separately, the constant-slope charging principle—precharge a capacitor to a code-dependent voltage then discharge at a fixed slope such that buffer nonlinearity is exercised identically at every code—is well established for DTCs, from resistive-DAC cores [12] through capacitor-DAC variants [13,14] and current-steering refinements to 250 MHz [15], including in the same 22 nm FDSOI process used here [16]. Uncalibrated, these cells reach 0.17% INL over a 10-bit range at up to 250 MHz [12,15]; the present work instead targets a 12-bit range from a mismatched 12-bit ladder at a 10 GHz input, where static bit-weight mismatch—not buffer nonlinearity—dominates the INL and must be measured out. Deterministic per-bit self-calibration has been standard in data converters for four decades, from Lee–Hodges–Gray [17] to modern SAR ADCs [18,19], but to our knowledge has not been transferred to the delay domain of an FOD DTC; that transfer is the core of this paper. Digital-to-phase converters sidestep delay-line INL entirely but presuppose a multi-phase spine and inherit phase-mismatch spurs, whereas the FOD here needs only a single input clock; with digital cancellation they reach near −106 dBc [20].

### 1.3. Contributions

This work contributes the following. First, it embeds a DAC-based constant-slope delay cell in a 10 GHz-input FOD. This combination had been considered unusable because the DAC settling time appeared to consume the whole timing budget; constant-slope DTCs reported to date operate at input rates of tens to hundreds of MHz [12,13,14]. We show that the delay range an FOD requires is only one period of the fast input clock, not of the divided output, and that allocating one input period to discharge and the remaining N−1 to DAC settling removes the bottleneck for practical division ratios. Second, the DAC is deliberately chosen as an R-2R ladder, against the capacitor-DAC trend of prior constant-slope work, because the ladder possesses a property no C-DAC offers: individual bit branches can be reconfigured (floated to the reference) without changing the Thévenin output resistance, so the settling behavior is invariant, and each reconfiguration maps one bit weight into a measurable delay shift. Based on this property, we build a direct INL self-calibration using radix weight extraction: a SAR-driven edge comparison measures the five mismatch-dominant (most significant) bit weights deterministically in a fixed and small number of steps, and a 32-entry segment look-up table indexed by those five bits linearizes all 4096 codes. In contrast to the statistical estimators of [1,2,11], the procedure has a deterministic step count and requires no decorrelation assumption; edge decisions near alignment, dithered by jitter, are averaged over a repetition count fixed at design time, so measurement noise enters as a budgeted variance rather than an unbounded convergence time; the scheme admits a complete error analysis. Third, we derive exact alignment conditions for the gain measurement, including a deliberate two-LSB code adjustment without which the measured gain would be biased by exactly one DTC LSB, and we prove geometric convergence of the coupled offset and gain loops. Fourth, we derive the exact class of INL errors the per-bit procedure cannot correct, namely the quadratic bow contributed by the finite output resistance of the discharge current source, in closed form, together with two mitigation paths; prior FOD INL calibrations absorb this class only empirically through their polynomial segments [2]. Fifth, we evaluate the calibrated divider at system level. A 200-die system-level Monte Carlo, anchored to the transistor-level mismatch statistics, gives the INL and DNL distributions, the FOD spur levels, and the deterministic jitter before and after calibration, together with the sensitivity of the procedure to comparator noise and offset, vernier nonlinearity, and calibration depth.

Three obstacles had to be overcome. (1) Any calibration that tunes the gain through a capacitor bank simultaneously shifts the zero-code offset; the offset and gain loops are therefore coupled, and we prove that the alternating calibration converges geometrically with a contraction factor equal to the product of the two coupling coefficients. (2) Local random mismatch decorrelates the main chain from the calibration chain; Monte Carlo analysis in the 22 nm FDSOI process placed the raw correlation at 0.49 once local mismatch was enabled, against 0.994 under global process variation alone. Tracking is restored to 0.94 by a Pelgrom-budgeted area allocation—a 32× DAC resistor-unit area and a 64× delay element, both at fixed nominal resistance and capacitance—which improves matching without altering the settling budget (Section 3). (3) At a 24.4 fs LSB, thermal noise, comparator noise, and above all power supply-induced jitter approach the LSB itself, so noise budgets had to be derived and respected throughout the design.

The constant-slope cell is linear by construction: with fixed $I_{\mathrm{dis}}$ and *C*, the delay is affine in the precharge voltage, which is in turn affine in the DAC code, so its error sources are few and separable. We organize the design around a classification of those sources by the treatment each admits (Table 1). Real-time random noise can only be traded against power and area; random mismatch, fixed at fabrication, is measured on chip and calibrated; slow drift from temperature and aging is tracked by periodic re-calibration; and errors that are both measurable and physically modeled are suppressed at the source by circuit design. On-chip measure-and-calibrate of the mismatch class is the central contribution of this work. Because every measurement is a time comparison between two nominally identical chains sharing process, supply, and temperature, the calibration needs no precision reference beyond the input clock itself; the specific per-error corrections are detailed in Section 4.

All results in this paper are obtained in the GlobalFoundries 22 nm FDSOI (22FDX) technology (GlobalFoundries Inc., Malta, NY, USA). The paper is organized as follows. Section 2 develops the FOD architecture and the timing scheme. Section 3 presents the delay cell and the DAC design trade-offs. Section 4 derives the calibration methodology. Section 5 reports the simulation results, and Section 6 discusses limitations. Section 7 concludes.

## 2. FOD Architecture and Timing Scheme

### 2.1. Required DTC Range

Figure 1 shows the FOD. The input clock at $f_{\mathrm{in}}=10$ GHz (period $T_{\mathrm{in}}=100$ ps) drives an MMD whose instantaneous division ratio toggles between *N* and N+1 under DSM control, so that the average ratio equals N+α with fractional word α∈[0,1). The DSM quantization error is canceled by delaying each divided edge with the DTC by

(2) $$t_{\mathrm{DTC}}\left[k\right]\;=\;\varepsilon_{q}\left[k\right]\;T_{\mathrm{in}},\;\varepsilon_{q}\left[k\right]\in \left[0,1\right),$$

where $\varepsilon_{q}\left[k\right]$ is the accumulated fractional residue at edge *k*. Equation (2) makes the central range requirement explicit: since $\varepsilon_{q}\left[k\right]<1$, the DTC full-scale range is exactly one input clock period,

(3) $$T_{\mathrm{FS}}\;=\;\frac{2^{B}-1}{2^{B}}\;T_{\mathrm{in}}\;\approx \;T_{\mathrm{in}},$$

and not one period of the divided output $T_{\mathrm{out}}=\left(N+\alpha \right)T_{\mathrm{in}}$. This observation is elementary but decisive. Earlier assessments of DAC-based delay cells assumed the cell had to span $T_{\mathrm{out}}$, concluded that the required voltage swing exceeded the DAC reference, and abandoned the architecture. With the range fixed at $T_{\mathrm{in}}=100$ ps and a B=12 bit DAC, the DTC resolution is

(4) $$T_{\mathrm{LSB}}\;=\;\frac{T_{\mathrm{in}}}{2^{B}}\;=\;\frac{100\;\mathrm{ps}}{4096}\;\approx \;24.4\;\mathrm{fs}.$$

### 2.2. Discharge/Settle Timing Allocation

The delay cell operates in two phases: the DAC charges the timing capacitor to the code-dependent voltage, and a constant current discharges it until an inverter threshold crossing produces the output edge. If both phases had to share one input period, a 12-bit settled precharge would be impossible at 10 GHz. They need not compete: within each divided output period of $N\;T_{\mathrm{in}}$, one input clock period is allocated to discharge and the remaining N−1 to DAC settling for the next edge (Figure 2). A control signal derived from the divided clock gates the discharge network for exactly one $T_{\mathrm{in}}$ regardless of code (fixed, not pulse-width modulated), and its delayed complement reconnects the DAC.

Modeling the DAC with its switch resistance as a first-order network of time constant $\tau =R_{\mathrm{th}}\;C_{\mathrm{tot}}$, where $R_{\mathrm{th}}=R$ for the R-2R ladder (code independent) and $C_{\mathrm{tot}}$ is the timing-node capacitance, the capacitor voltage at the end of the settling window $t_{s}=\left(N-1\right)T_{\mathrm{in}}$ is

(5) $$V_{C}\left(t_{s}\right)\;=\;V_{\mathrm{DAC}}\left(D\right)\;\left(1-e^{-t_{s}/\tau }\right)\;=\;V_{\mathrm{DAC}}\left(D\right)\;\left(1-\varepsilon_{s}\right),\;\varepsilon_{s}=\exp\;\left(-\frac{\left(N-1\right)T_{\mathrm{in}}}{\tau }\right).$$

Requiring the settling error to remain below one half LSB of the *B*-bit DAC gives the design inequality

(6) $$\varepsilon_{s}\;\leq \;2^{-(B+1)}\;⟺\;\frac{\left(N-1\right)\;T_{\mathrm{in}}}{\tau }\;\geq \;\left(B+1\right)\ln2\;\approx \;9.0\;\;\left(B=12\right).$$

With the ladder unit resistance R=50Ω and the timing-node capacitance $C_{\mathrm{tot}}=2$ pF in Section 3.4, $\tau =R\;C_{\mathrm{tot}}=100$ ps, and inequality (6) is satisfied for every N≥10. At N=10 the window provides exactly the 9.0 time constants the 12-bit budget requires; at N=20 (500 MHz output) it provides 19, and the settled accuracy exceeds the requirement by orders of magnitude. The unit resistance is the lever that sets this bound, and it trades against static current: doubling it to 100Ω would halve the ladder reference current from 8 to 4 mA (Section 5.8) but push the minimum ratio to N≥19, capping the output at 526 MHz.

Two properties rescue the small-*N* case. First, because $R_{\mathrm{th}}$ of the R-2R ladder is code independent, $\varepsilon_{s}$ in (5) multiplies $V_{\mathrm{DAC}}\left(D\right)$ uniformly across all codes: incomplete settling is a pure gain error, not a nonlinearity, provided the discharge phase fully resets the node each cycle. Second, a multiplicative voltage deficit is exactly absorbed by the gain calibration in Section 4: since the generated delay is $\Delta t=C\;\Delta V/I_{\mathrm{dis}}$, replacing *C* by

(7) $$C^{\prime }\;=\;\frac{C}{1-\varepsilon_{s}}$$

restores the nominal delay range. Because the settling is monotonic, code ordering is preserved and the per-code linearity survives gain calibration even with only a few time constants of precharge. The FOD reported here uses N≥10 and the fully settled budget of (6), where $\varepsilon_{s}<2^{-(B+1)}$ and the settling gain is constant to well below one LSB.

## 3. Constant-Slope Delay Cell Design

### 3.1. Operating Principle

The cell (Figure 3) realizes the constant-slope charging principle [12]: because the discharge always traverses the threshold with the same slope, the delay of the threshold buffer is exercised identically at every code, which is the structural reason constant-slope DTCs achieve intrinsically better INL than variable-slope delay lines [12,13]. During the settling window, the timing node is charged to $V_{\mathrm{start}}\left(D\right)=V_{\mathrm{DAC}}\left(D\right)$. At the divided clock edge the discharge current source $I_{\mathrm{dis}}$ is enabled, and the node ramps down with constant slope $I_{\mathrm{dis}}/C$; the output edge fires when the node crosses the threshold $V_{\mathrm{TH}}$ of the following inverter. For an ideal current source,

(8) $$t_{d}\left(D\right)\;=\;t_{0}\;+\;\frac{C\;\left(V_{\mathrm{start}}\left(D\right)-V_{\mathrm{TH}}\right)}{I_{\mathrm{dis}}}\;=\;t_{0}^{\prime }\;+\;\underset{G}{\underset{︸}{\frac{C\;V_{\mathrm{ref}}}{2^{B}\;I_{\mathrm{dis}}}}}\;D,$$

where $t_{0}^{\prime }$ collects all code-independent propagation delays and *G* is the DTC gain (seconds per LSB). Equation (8) exhibits the two independent tuning handles used throughout this work: the capacitance *C* scales the gain, and the code *D* scales the delay linearly. Deliberately using both simultaneously as signal inputs would produce a multiplicative, hence nonlinear, characteristic; therefore the code drives only $V_{\mathrm{start}}$, and *C* is reserved for calibration.

Compared with inverter-chain DTCs, the constant-slope cell has three structural advantages at femtosecond resolution. The step size is not quantized by a gate delay (10 to 15 ps for an inverter in this technology, more than two orders of magnitude coarser than the 24.4 fs target). Jitter does not accumulate across hundreds of stages, because a single ramp generates the entire delay. Only one node toggles at full swing, limiting dynamic power. Unlike a phase interpolator, which requires two bounding edges, the constant-slope cell places a delayed edge from a single incoming edge. Figure 4 shows the simulated cell in operation: the output edge displaces proportionally to the DAC code, with the code-0 and full-code traces bracketing the one-input-period range of Equation (3).

### 3.2. Linearity of the Ramp: Built-In Predistortion

In 22 nm FDSOI, neither the load capacitance nor the discharge current is constant along the ramp. The dominant load is the gate of the threshold inverter, which behaves as a MOS varactor: its capacitance falls from $C_{\max}=254$ fF at V=0.8 V to 165 fF at V=0.4 V, a droop of ΔC=89 fF (35%), while the discharge device simultaneously loses drive as its drain voltage drops. Linearity of the slope, however, requires only that the ratio I(V)/C(V) remain constant along the ramp, not that each term be constant individually. Writing the local slopes as $\lambda_{C}=\left(1/C\right)\;\partial C/\partial V$ and $\lambda_{I}=\left(1/I\right)\;\partial I/\partial V$, the design condition is

(9) $$\frac{d}{dV}\;\left(\frac{I(V)}{C(V)}\right)=0\;⟺\;\lambda_{I}\;=\;\lambda_{C}.$$

The varactor thus provides built-in predistortion: as the node voltage falls, the shrinking capacitance accelerates the ramp by the same factor by which the weakening current source decelerates it. The strong 22 nm varactor slope is diluted by a fixed metal capacitor $C_{0}$ in parallel, and the channel length and width of the discharge device are tuned so that (9) holds across the used voltage window; $C_{0}$ also serves as a mismatch compensation handle. The precharge PMOS is sized at 5 μm width so that the heavy node returns to $V_{\mathrm{start}}$ within the allocated settling window without slew limiting. The residual curvature that this predistortion leaves, and its suppression at the source by the regulated cascode, are derived in closed form and quantified at transistor level in Section 4.7.

### 3.3. DAC Design Space: Why the Obvious Choices Fail

The DAC is the precision-limiting block, and its failure modes are instructive.

Prior constant-slope DTCs generate $V_{\mathrm{start}}$ predominantly with capacitor DACs [13,14], which are attractive for power but offer no mechanism to isolate individual bit weights after fabrication: charge redistribution entangles all capacitors in every code. The R-2R ladder is retained here specifically because Section 4.5 exploits its bit-level reconfigurability for calibration. That choice must survive the ladder’s known failure modes, examined next; Table 2 summarizes how the alternatives compare.

*Resistor tolerance enters as gain, not as nonlinearity.* Integrated polysilicon resistors carry absolute tolerances of tens of percent and temperature coefficients of hundreds of ppm/°C, which is the usual objection to resistive DACs. In a voltage-mode R-2R ladder neither quantity reaches the INL. Every output voltage is a ratio of resistances, so a common sheet resistance or temperature shift cancels from $V_{\mathrm{DAC}}\left(D\right)/V_{\mathrm{ref}}$ exactly; what remains of it (through the reference current and the settling time constant $\tau =R\;C_{\mathrm{tot}}$) is common to all codes and is removed by the gain calibration in Section 4.3, or by the periodic refresh in Section 6 when it drifts. What sets the INL is the random ratio mismatch between nominally equal units, whose standard deviation follows the Pelgrom law $\sigma \left(\Delta R/R\right)=A_{R}/\sqrt{WL}$ [21] and is reduced here by the 32× unit area. That residue is precisely the quantity the radix extraction in Section 4.5 measures and cancels, so resistor tolerance stops being the limiting specification: the system-level Monte Carlo in Section 5.7 reproduces the ±13 LSB transistor-level INL envelope with a unit-resistor ratio mismatch of 0.42%, and the calibration removes it. The voltage coefficient of the resistors produces a smooth, static nonlinearity over the 0.4 V ladder swing; it belongs to the curvature class in Section 4.7 rather than to the per-bit class.

*Switch resistance kills R-2R linearity at speed.* Each ladder bit switch contributes an on-resistance $R_{\mathrm{on}}$ in series with its 2R rung. The resulting voltage error is code dependent because $R_{\mathrm{on}}$ varies with the switch operating point, so it appears as INL rather than as a benign gain term. Preserving 12-bit static linearity requires $R_{\mathrm{on}}\ll R/2^{B+1}$; for practical ladder resistances this demands switch resistances three to four orders of magnitude below what a minimum-overdrive CMOS switch achieves. Widening the switches restores $R_{\mathrm{on}}$ but multiplies the switch parasitic capacitance in proportion, and the product $R_{\mathrm{on}}\cdot C_{\mathrm{sw}}$ is a technology constant: brute-force sizing cannot win both terms. In this design, the conflict is resolved by three measures: the ladder unit resistance is chosen so that $10\;R_{\mathrm{on}}\leq R$, the switches use bootstrapped gate drive to make $R_{\mathrm{on}}$ signal independent, and, critically, the residual code-dependent error is left to the INL calibration in Section 4.5, which measures and corrects exactly this class of static per-bit error. The dynamic residue that static trimming cannot see was bounded directly: an eleven-point transient code sweep with the real bootstrapped switches, repeated with ideal switches and with the static per-bit fit subtracted, leaves a dynamic INL residue peaking at 0.42 LSB near mid-scale and an ascending-versus-descending hysteresis within ±0.13 LSB, confirming that the $\left(N-1\right)T_{\mathrm{in}}$ settling window erases code history. Both sit inside the 0.5 LSB dynamic budget.

*Internal ladder settling adds hidden delay.* Each internal ladder node settles with its own time constant; the slowest pole, not the output pole, bounds the usable conversion rate. This is accounted for in the budget of (6) by taking τ as the dominant measured time constant of the extracted ladder rather than the nominal $R\;C_{\mathrm{tot}}$.

*Matching sets device area, not resistance value.* The 32× enlargement of the DAC unit resistor (Section 5) increases device area at fixed resistance *R*: since the Pelgrom mismatch variance scales inversely with device area, a 32× area improves resistor matching by $\sqrt{32}\approx 5.7\times$, restoring the chain tracking reported in Section 5.Because *R* and the node capacitance are unchanged, the Thévenin time constant $\tau =R\;C_{\mathrm{tot}}$ and the settling budget of (6) are unaffected: the sizing improves matching without altering the N≥10 operating condition.

Two conventional mismatch remedies were considered and rejected. Partially thermometer-coding the MSBs eases the calibration burden [2] but multiplies switch count and routing parasitics on the settling-critical node and surrenders the bit-level reconfigurability that Section 4.5 depends on—buying a mismatch reduction the calibration already provides. Dynamic element matching is likewise unattractive: scrambling the ladder switches injects code-dependent activity into the settling window, aggravating the dynamic errors bounded above and converting deterministic spurs into a raised noise floor rather than removing the error.

**What calibration can and cannot fix.** Foreground calibration corrects static, repeatable, code-dependent errors. It cannot correct dynamic errors (switching transients, code-history effects) or environment-dependent drift. The design rule adopted here is therefore: size and bootstrap the DAC until its residual errors are static and below 0.5 LSB at an intermediate accuracy level, then let calibration bridge the remaining gap to 12-bit accuracy.

### 3.4. Noise and Supply Sensitivity Budget

This section addresses the random noise class (Class 1 of Table 1): errors that cannot be measured or calibrated and can only be traded against power and area. At $T_{\mathrm{LSB}}=24.4$ fs, random noise must be budgeted explicitly. The delay sensitivity to any voltage disturbance $\sigma_{V}$ at the threshold-crossing instant is set by the ramp slope $I_{\mathrm{dis}}/C$:

(10) $$\sigma_{t}\;=\;\sigma_{V}\;\frac{C}{I_{\mathrm{dis}}}.$$

Three contributors follow from (10).

*Thermal noise on the capacitor.* The sampled noise is $\sigma_{V}=\sqrt{kT/C}$, so

(11) $$\sigma_{t,\mathrm{th}}\;=\;\frac{\sqrt{kT\;C}}{I_{\mathrm{dis}}}.$$

At a fixed ramp slope $I_{\mathrm{dis}}/C$ (hence fixed delay gain, so $I_{\mathrm{dis}}\propto C$), $\sigma_{t,\mathrm{th}}\propto 1/\sqrt{C}$; Equation (11) therefore sets a lower bound on *C* that grows with the required resolution, which is the dominant constraint at this LSB. For the design point C=2 pF and $I_{\mathrm{dis}}=8$ mA, $\sigma_{V}\approx 45\;\mu$V and $\sigma_{t,\mathrm{th}}\approx 11$ fs, just below one half DTC LSB.

*Threshold-detector noise.* The inverter input-referred noise adds through the same (10); with the 4 V/ns ramp of the design point, each 100 μV of detector noise contributes 25 fs, so the inverter chain is sized for an input-referred noise below ∼10μV, contributing ≈2 fs—well under the thermal floor and consistent with the comparator entry of Figure 5.

*Power supply-induced jitter (the dominant killer).* The inverter threshold moves with the supply, giving a simulated sensitivity of approximately 0.25 ps per mV of supply deviation. One millivolt of supply noise therefore produces ten LSBs of jitter. Keeping the supply contribution below one LSB requires $\sigma_{V_{\mathrm{DD}}}≲100\;\mu V$ rms at the threshold inverter, which mandates a dedicated LDO for the ramp-and-detector core and aggressive local decoupling. This budget, not linearity, dictated the floorplan of the cell. Figure 5 places the three contributors against the LSB.

### 3.5. Fine-Delay (Calibration) Chain

Calibration requires a second, nominally identical delay chain (the calibration chain, CC) against which the main chain (MC) is compared, plus a fine-delay (FD) tuner inside the CC. Following the naming convention adopted for this design, the MC resolution is the *DTC LSB*, $T_{\mathrm{LSB}}\approx 24.4$ fs, and the CC tuning resolution is the *calibration LSB*,

(12) $$T_{\mathrm{CAL}}\;=\;\frac{1}{2}\;T_{\mathrm{LSB}}\;\approx \;12.2\;\mathrm{fs}.$$

A quarter-LSB granularity was considered and rejected because it doubles the FD DAC demand for a marginal accuracy return; all convergence targets below are expressed against the half-LSB granularity of (12).

Implementing the FD by switching small capacitors proved too nonlinear in this technology, because the switch parasitics are comparable to the intended unit capacitance. The FD is instead realized by binary weighting of the discharge transistor width,

(13) $$W_{\mathrm{eff}}\;=\;W_{0}+\sum_{i=0}^{N_{F}-1}b_{i}\;2^{i}\;W_{\mathrm{unit}},\;t_{\mathrm{FD}}\;\propto \;\frac{C_{\mathrm{fixed}}+C_{\mathrm{db},0}+\sum_{i}b_{i}2^{i}C_{\mathrm{db},\mathrm{unit}}}{W_{\mathrm{eff}}},$$

where the numerator collects the fixed load and the code-dependent drain parasitics. The parasitic term in the numerator partially tracks the denominator, which linearizes the tuning characteristic; the residual FD nonlinearity is uncritical because the SAR uses the FD only as a comparison vernier, never as a signal-path element. In the transistor-level runs, the phase comparison is represented by an *ideal comparator model*, a decision element returning the sign of the difference between the MC and CC edge times; the SAR engine and the binary-to-thermometer mapping are likewise ideal (see the validation-scope statement in Section 5). The physical element intended for this decision is the transistor-level D flip-flop of the companion divider [22]. The accuracy argument does not rest on the comparator being ideal, because each of its three non-idealities enters the procedure differently. (i) A static timing offset $t_{\mathrm{os}}$ cancels exactly: every stored calibration quantity is the difference of two alignments made with the same comparator (the offset alignment against the gain alignment in Equation (17), and the two members of each carry pair in Equation (24)), so $t_{\mathrm{os}}$ drops out as the common $T_{\mathrm{os}}$ does and only consumes fine-delay range. (ii) Random input-referred noise $\sigma_{c}$ adds in quadrature to the jitter of the two compared edges and sets the repetition depth, $M_{0}=\left\lceil \left(2\sigma_{t}^{2}+\sigma_{c}^{2}\right)/\sigma_{\mathrm{tgt}}^{2}\right\rceil$ with $\sigma_{\mathrm{tgt}}=T_{\mathrm{CAL}}/8$ (Section 4.6); it lengthens the calibration but does not limit its resolution, which is fixed by the $T_{\mathrm{CAL}}=12.2$ fs vernier step. (iii) Metastability is a resolution-time question: the calibration reads each decision one divided period, $NT_{\mathrm{in}}\geq 1$ ns, after the edges arrive, hundreds of times the picosecond-scale regeneration time constant of a CMOS latch in this process, and a decision that is still unresolved behaves as one more noisy vote. Section 5.7 quantifies (i) and (ii). The SAR engine is asynchronous; because a SAR comparison needs only a monotonic accept/reject decision per step, the accuracy of the SAR clock itself is immaterial, and the divided output clock can safely clock the engine even while it is being calibrated.

## 4. Foreground Calibration Methodology

The calibration proceeds in three stages: offset alignment, gain measurement, and INL extraction. All three reuse the same hardware: the CC, the FD vernier, the phase comparator, and the SAR engine. Throughout this section, the MC transfer characteristic is modeled as

(14) $$T_{\mathrm{MC}}\left(D\right)\;=\;T_{\mathrm{os}}+G\;D,\;D\in \left\{0,\ldots ,2^{B}-1\right\},$$

and the CC as $T_{\mathrm{CC}}\left(F\right)=T_{\mathrm{cs}}+g\;F$ with nominal g=G/2 per (12). Two views organize the section: Figure 6 maps each impairment class to its correction, and Figure 7 gives the procedural sequence; both are derived stage by stage below.

**Figure 6:**
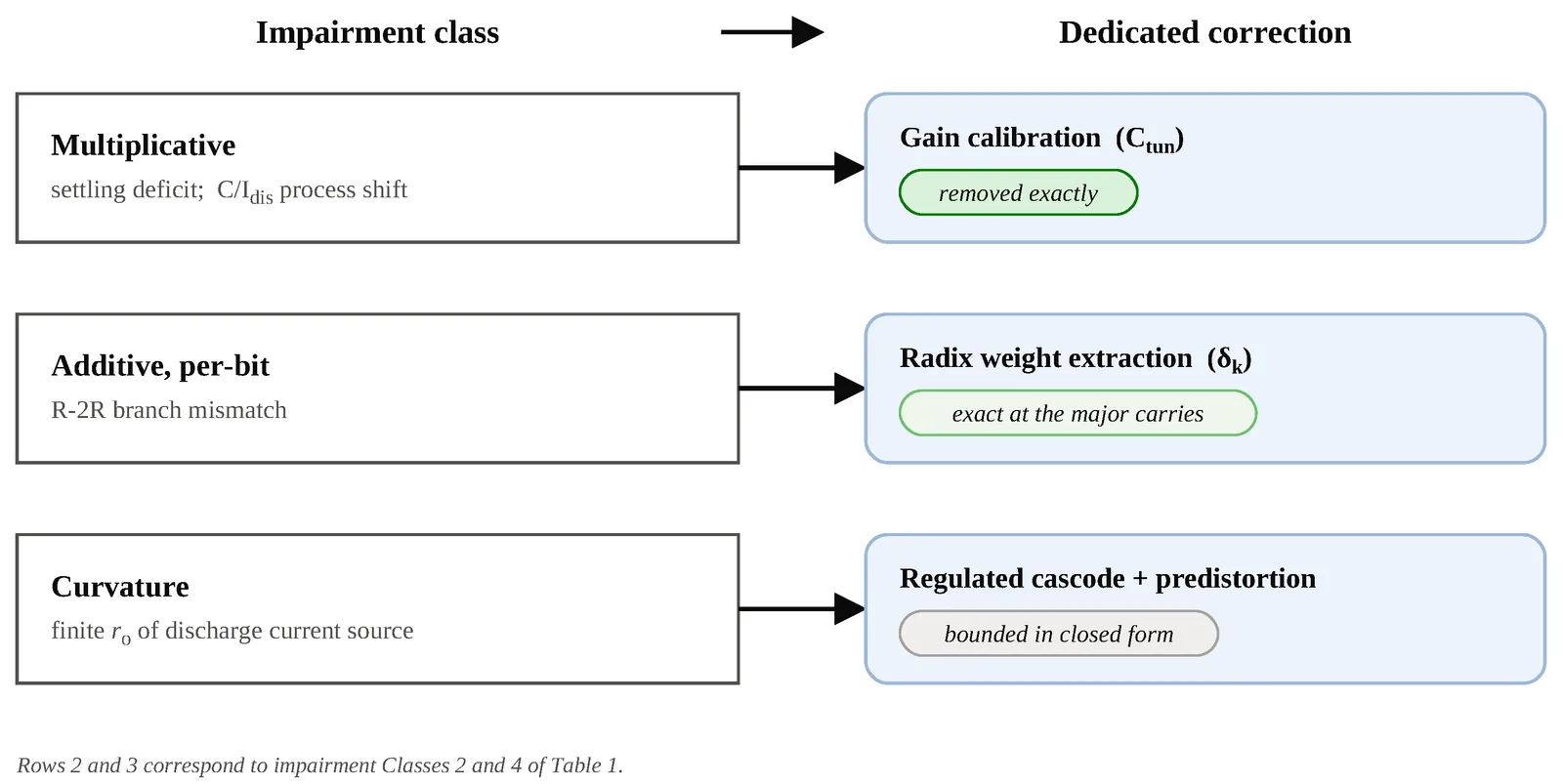
The three impairment classes of the constant-slope DTC and the dedicated correction assigned to each. Gain calibration (Section 4.3) removes its target exactly; radix extraction (Section 4.5) removes the per-bit error up to the measurement and table-step bounds in Section 5.7; the curvature term, which the calibration cannot observe, is bounded in closed form (Equations (29) and (30)) and suppressed at the source.

**Figure 7:**
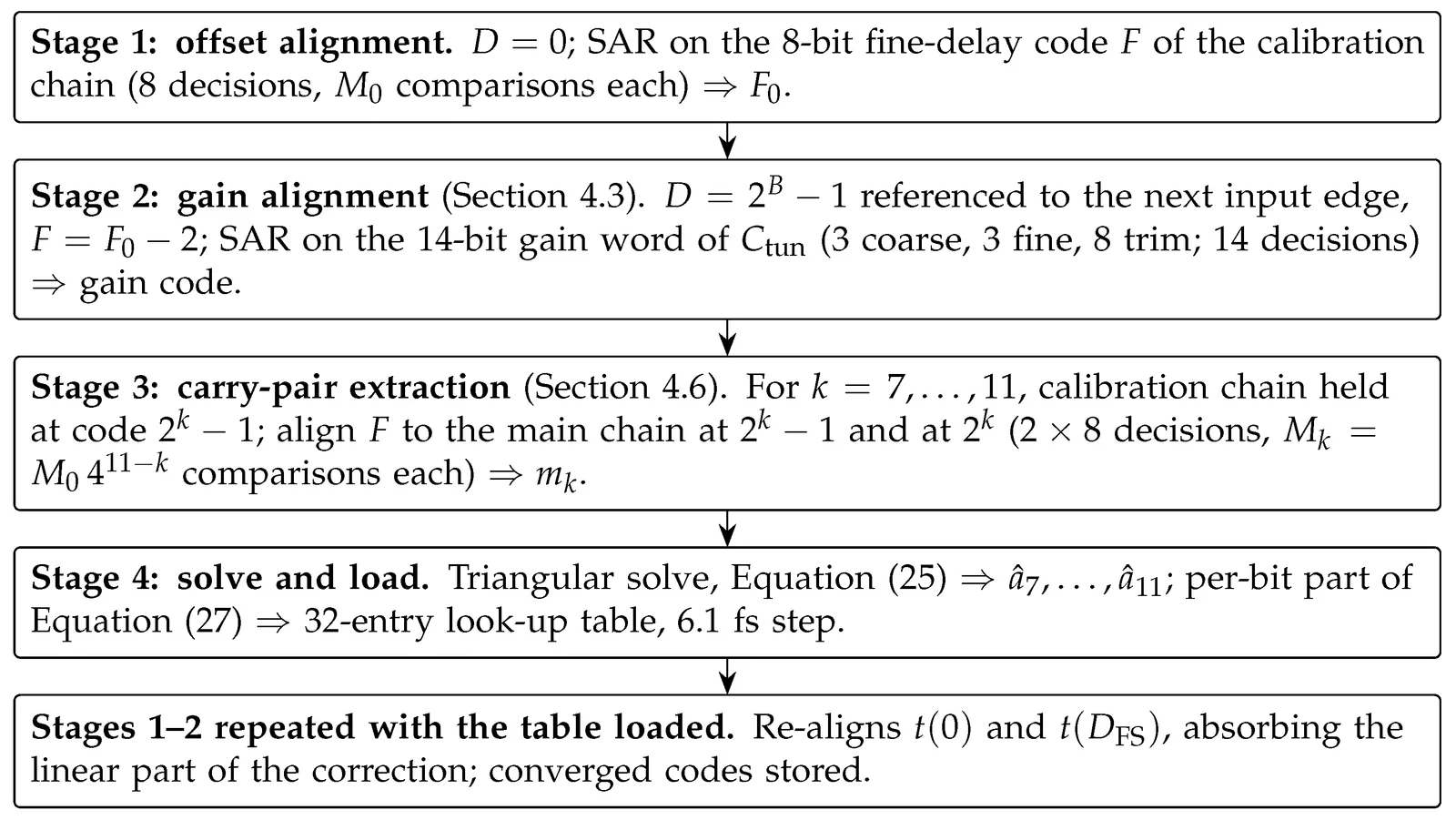
Foreground calibration sequence. Stages 1–2 form the coupled offset/gain loop in Section 4.4; its first iteration precedes the extraction, and its second, run with the look-up table loaded, converges the codes and absorbs the linear part of the correction. Stage 3 measures the five MSB weights by carry-pair alignments under the repetition schedule. All stages reuse the same hardware and need no reference beyond the input clock; code widths and decision counts are listed in Table 3.

**Table 3:** Calibration codes and decision counts. $M_{0}=108$ comparisons per decision at the thermal noise floor (Section 4.6).

Stage	Adjusted Element; Comparison	Code	Decisions	Repetitions
1 Offset	fine-delay vernier of the CC; MC at D=0 vs. CC	*F*, 8-bit	8	$M_{0}$
2 Gain	$C_{\mathrm{tun}}$ of the MC; MC at $D_{\mathrm{FS}}$ vs. CC at $F_{0}-2$ on the next edge	14-bit word: 3 coarse, 3 fine, 8 trim	14	$M_{0}$
1–2 repeated	as above, look-up table loaded	as above	22	$M_{0}$
3 Extraction	fine-delay vernier; MC at $2^{k}-1$ and at $2^{k}$ vs. CC held at $2^{k}-1$, k=7,…,11	*F*, 8-bit	5×2×8	$M_{0}\;4^{11-k}$
4 Correction	32-entry table indexed by $b_{11}\ldots b_{7}$, driving the auxiliary delay element	8-bit, 6.1 fs step	—	—
Total			124	$N_{\mathrm{cmp}}=5500\;M_{0}$

### 4.1. Error Partitioning: Gain Versus INL

Uncalibrated gain error is dominated by global process variation (systematic shifts of sheet resistance, oxide thickness, mobility) and must be budgeted at ±20 to 30% of full scale. Uncalibrated INL is dominated by local random mismatch between nominally identical devices and is budgeted at ±1 to 5%. This partition drives the hardware: the gain loop tunes a capacitor bank $C_{\mathrm{tun}}$ on the MC, with a coarse section for the process spread and a fine section for the final alignment, while the INL correction operates on a fine per-segment delay element. Local mismatch is first reduced by area (Pelgrom scaling [21]) until it sits inside the 1 to 5% window, and only the residue is calibrated.

### 4.2. Step 1: Offset Alignment at Code Zero

With D=0, the SAR adjusts the FD code until the comparator reports alignment between the MC output edge and the CC edge referenced to the same input clock edge. Only one polarity of edge (here the rising edge) is used for every comparison, because the input duty cycle is not trusted. The converged code $F_{0}$ satisfies

(15) $$T_{\mathrm{os}}\;=\;T_{\mathrm{cs}}+g\;F_{0}.$$

The quantity $F_{0}$ is the offset calibration result, stored with $T_{\mathrm{CAL}}$ granularity.

### 4.3. Step 2: Gain Measurement with the Two-LSB Correction

At full code, the MC delay increases by $(2^{B}-1)\;G$, which for a perfectly calibrated gain equals $\frac{2^{B}-1}{2^{B}}T_{\mathrm{in}}$: one DTC LSB short of a full period. The CC, when re-referenced to the next input clock edge, moves by exactly one full period $T_{\mathrm{in}}$. Naively aligning these two edges therefore forces a systematically wrong gain (Figure 8).

Before the full-code SAR search, the FD code is deliberately retreated by two calibration LSBs, $F=F_{0}-2$, which by (12) equals exactly one DTC LSB. The SAR then tunes the gain control (which scales *G*) until alignment. The capacitor bank $C_{\mathrm{tun}}$ has three sections: coarse and fine sections of three bits each, built from 3 fF units, which together span ±4.8% of the timing-node capacitance in steps that move the full-scale delay by 150 fs; and an 8-bit trim section of 0.2 fF units. Since those sections span ±4.8%, the remainder of the ±20 to 30% process spread has to be taken up by the discharge current itself, which the current-control block of Figure 1 sets. The smallest step sets the converged gain error, so it must satisfy

(16) $$\frac{\Delta G}{G}\;\leq \;\frac{T_{\mathrm{CAL}}}{T_{\mathrm{in}}}\;=\;1.2\times 10^{-4},$$

which is 0.24 fF referred to a 2 pF timing node. The 0.2 fF trim step meets it with margin, at $\Delta G/G=1.0\times 10^{-4}$; the 3 fF sections alone would not, and a step that coarse leaves a proportional gain error on every output edge, whose cost in the output spectrum is quantified in Section 5.7. The alignment condition is

(17) $$T_{\mathrm{os}}+G\;\left(2^{B}-1\right)\;=\;T_{\mathrm{in}}+T_{\mathrm{cs}}+g\;\left(F_{0}-2\right).$$

Subtracting (15) from (17) and substituting g=G/2:

(18) $$G\;\left(2^{B}-1\right)\;=\;T_{\mathrm{in}}-2g\;=\;T_{\mathrm{in}}-G\;⟹\;G\;=\;\frac{T_{\mathrm{in}}}{2^{B}}.$$

Equation (18) shows that with the two-LSB retreat, the converged gain equals the ideal $T_{\mathrm{in}}/2^{B}$ exactly, with no approximation. Without the retreat, the same algebra yields $G=T_{\mathrm{in}}/\left(2^{B}-1\right)$, a gain biased high by the factor $2^{B}/\left(2^{B}-1\right)$, i.e., by exactly one LSB accumulated across full scale. At 12 bits the bias (24.4 fs at full code) is comparable to the entire target accuracy, so the correction is not pedantic; at lower resolutions it would be fatal. The proof assumes g=G/2 exactly, whereas the FD is an independently mismatched chain; writing g=(G/2)(1+δ) and repeating the subtraction gives $G\;2^{B}=T_{\mathrm{in}}-G\delta$, hence $G=T_{\mathrm{in}}/\left(2^{B}+\delta \right)$: a fractional calibration LSB error δ perturbs the converged gain only by $\delta /2^{B}$, an attenuation of 4096×, so the measurement is self-immunized against FD gain error. The residual gain uncertainty after convergence is therefore set by the FD step and, provided the fine gain trim meets the requirement of Equation (16), is $\pm T_{\mathrm{CAL}}/2\approx \pm 6$ fs referred to full scale.

### 4.4. Step 3: Offset–Gain Coupling and Iterative Convergence

Tuning $C_{\mathrm{tun}}$ in Step 2 changes not only the slope *G* but also the zero-code delay $T_{\mathrm{os}}$, because the capacitor bank loads the timing node at all codes. After Step 2, the Step 1 alignment no longer holds, and repeating Step 1 disturbs Step 2 in turn.

Let α denote the offset shift produced per unit of gain correction and β the gain shift produced per unit of offset correction; both are small dimensionless sensitivities extracted from parametric simulation (representative value α≈0.1, i.e., 1 ps of offset shift per 10 ps of gain-range correction). One full iteration (offset phase followed by gain phase) maps the error pair $\left(E_{\mathrm{off}},E_{\mathrm{gain}}\right)$ to residuals attenuated by the loop product. After *n* iterations,

(19) $$E\left(n\right)\;=\;k^{\;n}\;E\left(0\right),\;k\;=\;\alpha \;\beta \;<\;1,$$

so the alternating calibration is a contraction mapping with geometric convergence. The number of iterations needed to reach a target residual $E_{\mathrm{target}}$ (here one half calibration LSB, 6.1 fs) is

(20) $$n\;\geq \;\left\lceil \frac{\ln\;\left(E_{\mathrm{target}}/E\left(0\right)\right)}{\ln k}\right\rceil .$$

For the extracted sensitivities of this design, $k\approx 10^{-2}$ and one to two iterations suffice. Since the procedure runs once at startup (foreground), the iteration cost is immaterial unless temperature or aging drift forces re-calibration; Section 6 returns to this point.

### 4.5. Step 4: INL Self-Calibration by Radix Weight Extraction

This is the measure-and-calibrate class (Class 2 of Table 1) and the central contribution of the paper: the R-2R bit-weight mismatch, random but fixed at fabrication, is measured directly on chip and corrected, rather than reduced by brute-force area.

Gain calibration linearizes only the two endpoints. Existing FOD INL calibrations estimate the interior of the transfer characteristic statistically, through sign-LMS loops that require decorrelated redundant states [1,11] or an auxiliary PLL as spur reference [2]; a direct, deterministic measurement of DTC INL, bit by bit, in the delay domain, is the missing capability, and it is the central claim of this work.

The method transfers the deterministic bit-weight self-calibration philosophy of data converters [17,18,19] from the voltage domain to the delay domain, exploiting a structural property of the R-2R ladder: disconnecting the MSB branch from the code and tying it to $V_{\mathrm{ref}}$ converts the 12-bit DAC into an 11-bit DAC whose Thévenin output resistance is unchanged (Figure 9), so the settling behavior is preserved while the output voltage is re-interpolated between the remaining ladder and the reference:

(21) $$V_{\mathrm{cap}}\;=\;\frac{V_{\mathrm{DAC}}\;R_{R}+V_{\mathrm{ref}}\;R_{O}}{R_{O}+R_{R}},$$

where $R_{R}$ is the resistance of the redirected branch, $R_{O}$ the resistance looking back into the remaining ladder, and, by superposition at the interpolation node, each source is weighted by the opposite branch resistance. Repeating the reconfiguration bit by bit (12 to 11 bits, 11 to 10, and so on) produces a family of interpolation equations of the form (21) with known nominal ratios: in each configuration, driving the reduced code to zero and to full scale places $V_{\mathrm{cap}}$ at nominally known fractions of $V_{\mathrm{ref}}$ (near $V_{\mathrm{ref}}/2$, $3V_{\mathrm{ref}}/4$, and so on). Because the voltage sits near mid-scale in every configuration, the corresponding output edge sits near the same input clock edge, and the FD vernier measures the small displacement of that edge with $T_{\mathrm{CAL}}$ resolution. Each measured displacement, through the constant slope $I_{\mathrm{dis}}/C$, is a direct reading of the deviation of one branch resistance ratio from nominal. Writing the MC delay in per-bit weight form,

(22) $$t\left(D\right)\;=\;t_{0}+\sum_{k=0}^{B-1}b_{k}\left(D\right)\;w_{k},\;w_{k}\;=\;2^{k}G\;\left(1+\epsilon_{k}\right),$$

the procedure solves for all $\epsilon_{k}$ from the measured edge displacements, two equations per configuration. An equivalent time-domain view is the major carry transition test: forcing the code from $\underset{k}{\underset{︸}{01\ldots 1}}$ to $\underset{k}{\underset{︸}{10\ldots 0}}$ ideally moves the edge by exactly one DTC LSB, which the CC resolves as exactly two calibration LSBs; any excess or shortfall, read on the FD vernier, is $\epsilon_{k}$ expressed in calibration LSBs. Each alignment is repeated *M* times and the resulting fine-delay codes are averaged. The two compared edges carry independent thermal jitter $\sigma_{t}$ (Section 3.4), so each comparison sees $\sqrt{2}\;\sigma_{t}\approx 15.6$ fs of noise, more than half the vernier step; the jitter therefore acts as a dither, and the average resolves the alignment below one vernier step with standard deviation $\sqrt{2}\;\sigma_{t}/\sqrt{M}$. A majority vote would discard this information and leave a $\pm T_{\mathrm{CAL}}/2$ quantization error per alignment, which the triangular solve in Section 4.6 would then amplify. *M* is chosen so that the averaged noise stays below 1.5 fs, about one eighth of the vernier step and a factor-of-two margin on the quarter-step band (±0.125 DTC LSB) used as the extraction target. The step count remains fixed; only the per-step repetition depth depends on the noise floor.

Static branch mismatch enters (22) as a linear superposition of per-bit errors. Measuring *B* numbers therefore suffices, in principle, to characterize and correct the INL of all $2^{B}$ codes. In this design, only the five most significant weights carry mismatch well above the trim resolution after the sizing in Section 5, so the correction vector $\left\{\delta_{k}=-{\hat{a}}_{k}\right\}$ has five nonzero entries (k=7,…,11); the other seven entries of the 12-bit weight model are fixed at zero by design, and Section 5.7 quantifies the residual this truncation leaves. The five values are expanded into a 32-entry look-up table indexed by the five MSBs, one entry per code segment, at quarter-LSB (6.1 fs) granularity, and the entry of the active segment drives an auxiliary delay element in the main path that only adds delay (the common constant is absorbed by the offset alignment). One subtlety must be respected: adjusting bit *k* changes the delay of all codes with $b_{k}=1$, including the full-scale endpoint, so each trim re-tilts the endpoint-fit line by $\delta_{k}/D_{\mathrm{FS}}$ and the corrections must be computed jointly, by the triangular solve in Section 4.6, not greedily.

### 4.6. The Explicit Extraction System and Its Noise Propagation

The reconfiguration admits a compact closed form. Tying the top *m* bits to $V_{\mathrm{ref}}$ and driving the remaining B−m bits with the reduced code $D_{m}$ places the timing node, nominally, at

(23) $$V_{\mathrm{cap}}\left(m,D_{m}\right)\;=\;V_{\mathrm{ref}}\left(1-2^{-m}+\frac{D_{m}}{2^{B}}\right),$$

which follows by induction from (21) with $R_{R}=2R$ and $R_{O}=2R$ at each ladder node, using the standard property that the ladder presents a Thévenin resistance of *R* at every internal node. Equation (23) fixes every nominal measurement position in advance ($V_{\mathrm{ref}}/2$ for m=1 at $D_{m}=0$, $3V_{\mathrm{ref}}/4$ for m=2, and so on), and its full-code value $V_{\mathrm{ref}}\left(1-2^{-B}\right)$ is configuration independent, a useful self-check. Two clarifications make the measurement plan exact. First, because rail assignments do not alter the transfer ratios of a linear resistive network, the reconfigured state {top *m* bits tied high, lower bits at $D_{m}\}$ is electrically identical to the normal-operation code with the same bit pattern; the reconfiguration adds no new electrical state. Its purpose is operational: the measured branches are static during the SAR search, so no switching disturbance corrupts the comparison. Second, the FD vernier spans only ±1.6 ps, so only pairs of states whose delays differ by a few LSBs are directly comparable; absolute positions such as FS/2 are not referenced to the input-edge grid. The measurable primitive is therefore the major carry pair, whose members are nominally one DTC LSB apart:

(24) $$\Delta_{k}\;\equiv \;t\left(2^{k}\right)-t\left(2^{k}-1\right)\;=\;w_{k}-\sum_{j<k}w_{j},\;m_{k}\;\equiv \;\frac{\Delta_{k}}{G}-1\;=\;a_{k}-\sum_{j<k}a_{j},$$

where $a_{k}\equiv 2^{k}\epsilon_{k}$ is the absolute weight error of bit *k* in DTC LSBs. The system (24) is triangular with unit diagonal and solves ascending from the lowest calibrated bit *ℓ*:

(25) $${\hat{a}}_{k}\;=\;m_{k}+\sum_{j=\ell }^{k-1}{\hat{a}}_{j},\;k=\ell ,\ldots ,B-1.$$

In this design ℓ=7: the weights of the lower seven bits sit below the mismatch floor after the sizing in Section 5 and are taken as ideal, so five measurements calibrate the five MSBs, consistent with the 32-segment correction granularity. The back-substitution (25) amplifies measurement noise geometrically: substituting recursively gives ${\hat{a}}_{k}=m_{k}+\sum_{j<k}2^{\;k-1-j}m_{j}$; hence, for independent per-measurement noise $\sigma_{m}$,

(26) $$\mathrm{var}\left({\hat{a}}_{k}\right)\;=\;\sigma_{m}^{2}\left(1+\frac{4^{\;k-\ell }-1}{3}\right),$$

a factor of 86 (amplitude factor 9.3) at k−ℓ=4. The mitigation is a repetition schedule: measuring $m_{j}$ with $M_{j}\propto 4^{\;(B-1)-j}$ comparisons equalizes all contributions to the MSB estimate, and the total comparison count remains fixed and known at design time, preserving the deterministic step count property. Finally, the endpoint-preserving correction has a per-bit part and a linear part:

(27) $$c\left(D\right)\;=\;-\sum_{k}b_{k}\left(D\right)\;{\hat{a}}_{k}\;+\;\frac{D}{D_{\mathrm{FS}}}\sum_{j}{\hat{a}}_{j},$$

The first term cancels the per-bit superposition; the second is a pure gain term that keeps t(0) and $t(D_{\mathrm{FS}})$, and hence the calibrated gain, unchanged. The gain term depends on every bit of *D* and is therefore not stored: the second offset–gain iteration is run with the table loaded, which re-aligns both endpoints and absorbs it (Figure 7). The table holds only the first term, which depends on the five MSBs alone because ${\hat{a}}_{k}=0$ for k<ℓ and so fits exactly in 32 entries.

**Calibration cost.** The full foreground sequence has a fixed, design-time-known step count (Table 3). Offset alignment is an eight-step SAR search on the 8-bit fine-delay vernier, and the gain alignment a fourteen-step search on the capacitor word (3-bit coarse, 3-bit fine, 8-bit trim); the coupled loop in Section 4.4 converges in two iterations, the second run with the look-up table loaded, giving 2×(8+14)=44 SAR decisions. Radix extraction measures the five MSB weights; each carry pair needs two alignments, one per member, for 5×2×8=80 decisions, so the sequence is fixed at 124 SAR decisions independent of the mismatch realization. Only the per-decision repetition depth depends on noise: each decision averages $M_{0}$ comparisons in the offset and gain phases and $M_{j}=M_{0}\;4^{(B-1)-j}$ comparisons for the carry pair of bit *j*, so the total edge-comparison count is

(28) $$N_{\mathrm{cmp}}\;=\;\left[\;44\;+\;16\;\;\sum_{j=B-5}^{B-1}\;\;4^{(B-1)-j}\;\right]\;M_{0}\;=\;5500\;M_{0},$$

the extraction term ($16\times 341\;M_{0}$) dominating: the offset and gain searches together account for 44 of the $5500\;M_{0}$ comparisons. Every comparison involves two independent edges, so with the thermal edge jitter $\sigma_{t}\approx 11$ fs in Section 3.4 the per-comparison noise is $\sqrt{2}\;\sigma_{t}\approx 15.6$ fs, and the 1.5 fs averaging target requires $M_{0}=\left\lceil {\left(15.6/1.5\right)}^{2}\right\rceil =108$, so $N_{\mathrm{cmp}}\approx 5.9\times 10^{5}$. The delay cell fires once per divided period, so each comparison occupies one output period $NT_{\mathrm{in}}$: at the minimum ratio N=10 the complete foreground calibration takes ≈0.59 ms, and at N=20, ≈1.2 ms. A physical comparator with input-referred noise $\sigma_{c}$ raises $M_{0}$ in proportion to $2\sigma_{t}^{2}+\sigma_{c}^{2}$; at $\sigma_{c}=50$ fs the sequence lengthens to ≈6.7 ms at N=10 (Section 5.7). This is a one-time startup cost. The periodic refresh that temperature drift requires (Section 6) re-runs only the offset–gain loop, $44\;M_{0}\approx 4.8\times 10^{3}$ comparisons or 4.8μs at N=10.

### 4.7. What Per-Bit Trimming Cannot Fix: The Curvature Bound

The finite output resistance of the discharge current source is the exemplar of the modeled-error class (Class 4 of Table 1): measurable, described in closed form, and therefore best suppressed at the source by circuit design rather than measured out. The weight model (22) is exact only if the voltage-to-time conversion is itself linear. With a finite current-source output resistance the discharge current depends on the instantaneous node voltage, $I\left(v\right)=I_{0}\;\left(1+v/V_{A}\right)$, where $V_{A}=I_{0}\;r_{o}$ is the equivalent early voltage of the discharge stack. The delay integral then acquires a logarithmic form,

(29) $$t\left(V_{s}\right)=\int_{V_{\mathrm{TH}}}^{V_{s}}\frac{C\;dv}{I_{0}\;\left(1+v/V_{A}\right)}\;=\;\frac{C\;V_{A}}{I_{0}}\;\ln\;\frac{V_{A}+V_{s}}{V_{A}+V_{\mathrm{TH}}}\;\approx \;\frac{C}{I_{0}}\;\left[\left(V_{s}-V_{\mathrm{TH}}\right)-\frac{V_{s}^{2}-V_{\mathrm{TH}}^{2}}{2V_{A}}\right],$$

whose quadratic term converts the code-linear $V_{s}$ into a code-quadratic delay error. Expanding $V_{s}=\sum_{k}b_{k}2^{k}\Delta_{V}$ inside $V_{s}^{2}$ produces cross terms $b_{j}b_{k}$. No choice of per-bit trims $\delta_{k}$ can generate a $b_{j}b_{k}$ term, so per-bit trimming cannot cancel the quadratic error. The carry-pair test does not even respond to it: it measures only the local step at each major carry code, and a smooth parabola leaves those steps at their nominal value (its tangent at mid-scale is parallel to the endpoint chord). The full-range bow therefore survives calibration intact, as the transistor-level result of Figure 10e shows. Removing the endpoint-fit line from the quadratic term of (29) leaves a residual bow that peaks at mid-scale with magnitude

(30) $${\mathrm{INL}}_{\mathrm{bow}}\;=\;\frac{C\;V_{\mathrm{FS}}^{2}}{8\;I_{0}\;V_{A}}.$$

**Why the design still meets target.** Equation (30) identifies $V_{A}$ as the only free variable, and $V_{A}$ is a circuit choice, not a process given. A regulated (gain-boosted) cascode multiplies the output resistance of the stack by the loop gain, $r_{o}\to \left(A\;g_{m}r_{o}\right)\;r_{o}$, suppressing the bow by two to three orders of magnitude at the source; the low-voltage cascode biasing fits within the 0.8 V supply of the technology. The residue after cascoding, together with any higher-order terms, is smooth across the code range, and the self-referenced hardware cannot measure it: the only absolute time reference is the full input period, and the carry-pair measurements see only local steps, which a smooth curvature leaves at their nominal value. The residue therefore adds one-for-one to the calibrated INL (Section 5.7), and circuit design has to hold it below the INL target. This is why the four-class organization of Table 1 assigns it to suppression at the source rather than to calibration.

**Transistor-level confirmation.** The two transistor-level results that follow were obtained on the characterization testbench, whose bias differs from the design point in Section 3.4; Equation (31) below carries the result across. Figure 10 traces the major carry transition calibration stage by stage on the extracted delay cell. The gain-calibrated transfer curve exhibits a bit-weight INL of +30.0/−16.5 LSB; sequential trimming from MSB to LSB, re-run in LSB-to-MSB order to remove the stale reference residual, bottoms out at a +7.3/−0.0 LSB parabolic bow, exactly the residue that (30) predicts is orthogonal to the per-bit correction space. Figure 11 then sweeps the current-source output resistance $r_{o}$ (equivalently $V_{A}=I_{0}r_{o}$): the peak residual tracks the $1/r_{o}$ scaling of (30) precisely in the bow-dominated regime and saturates at a residual floor of order one LSB (the trim granularity plus residual noise) beyond it (Table 4).

The constant product $\mathrm{INL}\cdot r_{o}$ across the first three rows of Table 4 is the direct numerical confirmation of the $1/r_{o}$ law. What the sweep establishes is that scaling; its absolute scale belongs to the characterization testbench on which it was run, not to the design point. At the design point ($V_{\mathrm{FS}}=0.4$ V, $I_{\mathrm{dis}}=8$ mA, C=2 pF), Equation (30) with $V_{A}=I_{\mathrm{dis}}\;r_{o}$ gives

(31) $${\mathrm{INL}}_{\mathrm{bow}}\cdot r_{o}\;=\;\frac{2^{B}V_{\mathrm{FS}}}{8\;I_{\mathrm{dis}}}\;=\;2.6\times 10^{4}\;\Omega \cdot \mathrm{LSB},$$

so the bow falls below one LSB once $r_{o}>26\;$kΩ. Read against this relation, the +7.3 LSB residue of Figure 10e corresponds to an output resistance of about 3.5KΩ, the order a plain cascode reaches at 8 mA, while the regulated cascode multiplies $r_{o}$ by the loop gain and drives the residue two to three orders of magnitude below the LSB. The curvature class is therefore closed by circuit design at the design point, exactly as Table 1 assigns it.

## 5. Simulation Results

**Validation scope.** The contribution of this work is the calibration methodology, not the design of the surrounding building blocks. Accordingly, the elements that carry the claim—the constant-slope delay cell, the main and calibration delay chains, and the radix-weight INL extraction—are simulated at transistor level in the GlobalFoundries 22FDX PDK with Spectre, and all INL, tracking, and drift results below derive from those transistor-level runs. The standard supporting blocks that are not the contribution—the phase comparator, the SAR controller, and the binary-to-thermometer mapping—are represented by ideal Verilog-A models, so that the reported statistical behavior isolates the analog delay chains; Section 3.5 explains why the accuracy argument does not depend on this idealization, and Section 5.7 bounds its effect. The output spectrum of a fractional divider is a property of thousands of consecutive edges rather than of any single transient: resolving a −90 dBc spur on a 24.4 fs grid takes on the order of $10^{4}$ output periods per fractional word, across hundreds of mismatch samples. Those quantities—the FOD spectra, the spur levels, and the 200-die INL and DNL distributions after calibration—are therefore obtained by system-level Monte Carlo simulation driven by the transistor-level statistics above (Section 5.7), which is how spur families in this class of divider are normally predicted [23]. The circuit has not been fabricated. The flipped-well (forward-back-bias capable) device flavor is used in the delay cells.

### 5.1. Simulation Setup

Table 5 lists the conditions of the transistor-level runs, and the design parameters of every block are collected in Appendix A. Two conventions matter at this resolution and are fixed here. Delays are taken between threshold crossings at the 350 mV inverter trip point, with the simulator interpolating the crossing rather than reporting the nearest time step. And the LSB is defined as $T_{\mathrm{LSB}}=T_{\mathrm{in}}/2^{B}=24.4141$ fs (Equation (4)), not as the endpoint spacing $T_{\mathrm{in}}/\left(2^{B}-1\right)=24.4200$ fs; the two differ by 6 as, which is below every residual quoted in this paper but is stated so that reported INL figures can be reproduced exactly.

### 5.2. A Methodological Note on Monte Carlo Libraries

A practical note for this PDK: selecting “process only” Monte Carlo does not exclude local mismatch unless the library variant is switched to its no-mismatch counterpart. All process-only results below use the no-mismatch variant. The Monte Carlo studies that follow use 100 samples for the chain-tracking analysis, 200 for the offset–gain convergence sweep, and 31 for the temperature drift sweep. The PDK does not model gradient variation, which remains a layout-stage risk addressed by common-centroid placement of the two chains.

### 5.3. Main-Chain to Calibration-Chain Tracking

The entire calibration architecture rests on one statistical premise: the MC and CC must track under variation. Table 6 summarizes the correlation of the two chain delays over 100 Monte Carlo samples at the 100 ps nominal point, and Figure 12 shows the scatter before and after sizing.

Sensitivity analysis attributed the decorrelation primarily to DAC resistor mismatch and secondarily to the delay elements themselves, which motivated the 32× and 64× area increases, respectively. The residual ρ=0.94 spread is exactly the error class the calibration capacitors absorb, so no further area was spent.

### 5.4. Fine-Delay Range Dimensioning

The FD range must cover the worst-case deviation of the scatter points from the correlation line, on both sides, because at each process corner the FD must pull the CC delay back onto the line. That deviation follows directly from the post-sizing tracking statistics of Figure 12b. For two nominally identical chains of equal spread σ and correlation ρ, the chain-to-chain mismatch error $e=t_{\mathrm{MC}}-t_{\mathrm{CC}}$ has standard deviation

(32) $$\sigma_{e}\;=\;\sigma \sqrt{2\;(1-\rho )},$$

so the measured σ=1.5 ps and ρ=0.94 give $\sigma_{e}=0.52$ ps. Dimensioning the vernier to a $\pm 3\sigma_{e}$ design point, the required FD span is

(33) $$\frac{2\times 3\sigma_{e}}{12.2\;\mathrm{fs}/\mathrm{LSB}}\;=\;\frac{2\times 1559\;\mathrm{fs}}{12.2\;\mathrm{fs}/\mathrm{LSB}}\;\approx \;256\;\mathrm{calibration}\;\mathrm{LSBs}\;\Rightarrow \;8\text{-}\mathrm{bit}\;\mathrm{FD},\;\mathrm{mid}\text{-}\mathrm{code}\;\mathrm{centered},$$

where the 8-bit range (256 codes) meets the $\pm 3\sigma_{e}$ requirement exactly, so the vernier is dimensioned at its design point rather than with surplus. A 7-bit FD spans only ±780 fs, or $\pm 1.5\sigma_{e}$, and is therefore insufficient; conversely a $\pm 4\sigma_{e}$ target would require 341 codes and a 9-bit vernier, which is the trade available should a tighter yield bound be imposed. Because the fine delay need only null the chain-to-chain mismatch *e* rather than the absolute main-chain spread σ, the 8-bit range is sufficient at the stated design point. Engineering-corner simulations confirm the extreme codes are never exercised in operation.

### 5.5. Calibration Convergence

Across the 200-sample, seven-temperature sweep, the alternating offset/gain procedure in Section 4.4 converged below the 6.1 fs target (one half calibration LSB) within two iterations in all cases, consistent with the contraction bound (20) and the extracted $k=\alpha \beta \approx 10^{-2}$.

### 5.6. Linearity

Before calibration, the worst-corner INL is dominated by the endpoint gain error, consistent with the ±20 to 30% process budget. After offset and gain calibration but before per-bit correction, a representative sample exhibits 13.06 LSB peak-to-peak INL concentrated at the major carry codes (1024, 2048, 3072), the unmistakable signature of binary bit-weight mismatch, which is precisely the error class the radix extraction in Section 4.5 measures and removes; over all 200 mismatch samples the endpoint-fit envelope stays within approximately ±13 LSB (0.3% of full scale), inside the 1 to 5% mismatch budget and organized by the segment boundaries. This is the same class of major carry structure that the stepwise trimming of Figure 10 removes. The per-bit correction targets exactly this structure, and the extraction that feeds it has been validated in closed loop. Figure 13 shows a representative transistor-level run: the SAR-driven carry-pair measurements, processed through the triangular solve of Equation (25) with the repetition schedule active, recover all five injected MSB weight errors within the ±0.125 DTC LSB extraction band (one quarter of the vernier step) and with no growth across bit index—injecting known weight errors here isolates the triangular-solve estimator, the physical measurement fidelity being set separately by the edge-jitter budget in Section 3.4—confirming that the $M_{j}\propto 4^{(B-1)-j}$ schedule neutralizes the noise amplification of Equation (26); the uniform-repetition run in the same figure (Figure 13b, red) exhibits the predicted error growth toward the MSB, an independent empirical confirmation of that analysis. The end-to-end effect is a single chain (Figure 10 and Figure 11 in Section 4.7): offset and gain calibration correct the slope but leave the bit-weight INL, which for the high-mismatch cell of Figure 10 stands at +30.0/−16.5 LSB; per-bit radix trimming then reduces it to the +7.3 LSB curvature bow, and the regulated cascode finally drives it below one LSB by raising the current-source output resistance—each stage removing exactly the error class it targets.

The thermal noise floor of (11), 11 fs rms at the design point, sets the meaningful resolution limit of the residual-INL figures reported above.

### 5.7. System-Level Evaluation

The transistor-level runs above establish the delay cell, the chain tracking, and the extraction on individual dies. Three further quantities—the distribution of INL and DNL over many dies after calibration, the resulting FOD output spectrum, and the sensitivity of the procedure to its idealized comparator—are statistical and spectral properties of the complete divider, defined over thousands of consecutive output edges and hundreds of mismatch samples, and therefore outside the reach of any single transistor-level transient. They are obtained here by system-level Monte Carlo simulation (Python 3.11, Python Software Foundation, Wilmington, DE, USA; NumPy 2.1) whose device-level inputs come from the transistor-level results above and whose mechanics are first-principles: an exact nodal solution of the mismatched ladder, the SAR procedure executed comparison by comparison with the edge jitter in Section 3.4, and the delta-sigma edge sequence of the divider itself. The script and its data are provided as Supplementary Materials, so every number below is reproducible.

*Model.* Each die carries a 12-bit voltage-mode R-2R ladder whose unit resistors have independent Gaussian ratio mismatch; the ladder is solved by nodal analysis, which gives the exact bit weights and, by superposition, all 4096 delays. The unit mismatch, 0.42%, is the single fitted parameter: it is set so that the endpoint-fit INL envelope of 200 gain-calibrated dies equals the ±13 LSB of the transistor-level Monte Carlo. The calibration chain carries its own independent ladder and a chain-to-chain offset with $\sigma_{e}=0.52$ ps (Equation (32)). The calibration is executed as specified in Table 3. Every alignment is an 8-step SAR search on the fine-delay vernier in which each comparison sees the thermal jitter of both edges (11 fs each); the vernier carries a 2% step error and 5% rms per-code DNL; the searches are repeated and averaged with $M_{0}=108$ under the $M_{j}$ schedule; the weights follow from the triangular solve; and the correction is quantized to the 32-entry, 0.25 LSB table. The smooth V-to-t curvature is set to zero in the baseline and treated separately below, because the calibration cannot observe it (Section 4.7). The FOD is modeled at the edge level: 10 GHz input, N=10 (1 GHz output, the minimum ratio the settling budget in Section 2 admits), a first-order delta-sigma modulator whose 14-bit residue is truncated to the 12-bit DTC code, and 16,384 output edges per fractional word, which makes each record exactly periodic so that spur levels are read from a coherent FFT without windowing.

*INL and DNL.* Figure 14 shows the 200 dies before and after the per-bit correction. After gain calibration, the median peak INL is 4.1 LSB (95th percentile 7.8 LSB), organized at the major carry codes, and 83% of the dies are non-monotonic. After calibration the median peak INL is 0.32 LSB and the 95th percentile 0.54 LSB; 94% of the dies stay below 0.5 LSB and 199 of 200 below 1 LSB, with a median peak DNL of 0.28 LSB and every die but one monotonic. That die is not an extraction failure: its chain-to-chain offset exceeds the ±1.56 ps span of the 8-bit vernier, which the fine-delay dimensioning set at $\pm 3\sigma_{e}$ and so expects to miss a few dies per thousand; the 9-bit option discussed there removes it. The residual of the passing dies has three parts: the 0.25 LSB table step, the averaged measurement noise, and the uncalibrated lower seven bits. The 32-entry table needs 8-bit entries (1.56 ps range at a 6.1 fs step).

*Spurs and jitter.* Figure 15 propagates each die’s delay characteristic through the FOD for 23 fractional words between $6\times 10^{-5}$ and 0.5. Without calibration the gain error (±24% at 3σ) dominates and the median worst spur is −46.2 dBc. Gain calibration alone brings it to −75.5 dBc (95th percentile −68.9 dBc), in the range of the gain-calibrated FODs of Table 7. The per-bit correction brings every die to −91.3 dBc, the level of an ideal 12-bit DTC: the remaining tone, at $f_{\mathrm{out}}/4$, is the quantization-induced spur from truncating the 14-bit residue to the 12-bit code [23], which calibration cannot remove and dithering does. With the quantization error removed, the INL-induced worst spur falls from −75.5 dBc to −103.8 dBc (medians; 95th percentile −96.8 dBc after calibration). The deterministic jitter follows the same pattern: 53 fs rms (median over dies, worst fractional word) with gain calibration alone and 7.4 fs after calibration, against 6.8 fs for the ideal 12-bit DTC. Adding the random floor in Section 3.4 (26.8 fs with the supply term at budget) gives 60 and 28 fs rms of total edge jitter from the DTC. A smooth V-to-t bow of 1 LSB added after calibration raises the median peak INL to 1.24 LSB but moves the worst spur only to −90.1 dBc.

*Gain-step quantization.* The gain control enters the same spectrum through its own resolution, which is why Equation (16) is a requirement and not a preference. A residual relative gain error *q* leaves a sawtooth of amplitude $qT_{\mathrm{in}}$ on the output edges. Propagating it through the same model puts the implemented 0.2 fF trim section, whose half-step is $q=5\times 10^{-5}$, at −91.3 dBc: exactly the quantization floor, so the gain control contributes nothing measurable to the spectrum. Had the 3 fF sections been the finest step available, their half-step ($q=7.5\times 10^{-4}$) would have placed the floor at −82.5 dBc, some 9 dB higher. This is what Equation (16) buys, and why the trim section rather than the coarse bank sets the converged gain error.

*Sensitivity to the measurement hardware.* Figure 16 repeats the calibration on 60 dies while one non-ideality at a time is swept from the baseline. (a) Comparator noise: with $M_{0}$ scaled to the total noise as in Section 3.5, the post-calibration INL is unchanged up to 200 fs rms of comparator noise (median 0.35 LSB, 95th percentile 0.56 LSB), and the cost appears only in time, 98 ms at N=10 for 200 fs (panel f); with $M_{0}$ held at 108 the median degrades gradually, to 0.60 LSB at 200 fs. (b) Comparator offset: no effect up to 0.3 ps; at 1 ps the offset and the chain-to-chain mismatch together push 7 of 60 dies outside the ±1.56 ps vernier, so the offset budget of the comparator is a fine-delay range budget, not an accuracy budget. (c) Vernier nonlinearity: a carry-pair alignment exercises the vernier over only a few codes, and a per-code DNL of up to 10% of the step changes the median INL by less than 0.02 LSB; at 20% the 95th percentile reaches 0.8 LSB. (d) Calibration depth: calibrating four MSBs leaves a 0.42 LSB median, five (this design) 0.32 LSB, and six or more improve the median by only 0.03 to 0.07 LSB, as the 0.25 LSB table step and the measurement noise set the floor. (e) Curvature: the residual rises one-for-one with the full-scale bow (1.23 LSB median for a 1 LSB bow), confirming that the carry-pair measurement is blind to it and that it has to be held down at the source (Section 4.7).

### 5.8. Implementation Overhead

The calibration time is fixed by Equation (28): 0.59 ms at the minimum ratio N=10 and 1.2 ms at N=20 at the thermal noise floor, growing with comparator noise as in Figure 16f, while a temperature refresh costs 4.8 μs. The delay cell draws its discharge current for at most one input period per output period, so its core dissipates at most $I_{\mathrm{dis}}T_{\mathrm{in}}f_{\mathrm{out}}V_{\mathrm{DD}}\approx 0.64$ mW at $f_{\mathrm{out}}=1$ GHz. The ladder adds a static, code-dependent reference current of order $V_{\mathrm{ref}}/R=8$ mA at R=50Ω, i.e. up to 6.4 mW drawn from the 0.8 V supply, and the calibration chain, comparator, and SAR engine operate only during calibration and refresh and can be power-gated otherwise. The digital overhead is an 8-bit SAR, a 15-bit comparison counter, and a 32×8-bit table (Appendix A). Area is not reported because the circuit has not been laid out; the dominant analog items are the 2 pF timing capacitance and the 32× resistor array.

### 5.9. Positioning Against the State of the Art

Table 7 situates this work among recent FOD calibrations, and Table 8 among constant-slope DTCs. The upper part of Table 7 compares the calibration mechanism—estimation principle, convergence character, INL coverage, and whether a residual bound exists—which is where the contribution lies. The lower part adds the performance figures each work reports, with the validation type stated, because the prior FODs are measured silicon while the results here are simulated; values for the prior works are those stated in their abstracts, and n/r marks a figure not reported there.

## 6. Discussion and Limitations

**Layout dependence.** All transistor-level results are schematic-level with extracted-dominant-pole DAC modeling; layout parasitics are not included, and the circuit has not been fabricated. Three items are most sensitive to layout. (i) Chain tracking: the ρ=0.94 of Table 6 assumes purely random mismatch. A process gradient across the two chains adds a systematic offset, which the fine delay absorbs only up to its ±1.56 ps range, so the main and calibration chains must be interdigitated in a common-centroid arrangement, with dummy units around the 32× resistor array. (ii) Settling: routing capacitance on the timing node adds to $C_{\mathrm{tot}}$ and lengthens τ in Equation (5), so the $\left(N-1\right)T_{\mathrm{in}}$ window must be re-checked on the extracted netlist. (iii) Supply-induced jitter: the 100 μV budget in Section 3.4 has to hold with the package and the on-chip supply network included, discussed next.

**Path to silicon validation.** In the target SoC the 10 GHz input comes from the on-chip integer-N PLL and never crosses a package boundary, so the impedance matching that a test chip needs is a property of the test setup, not of the architecture. A test chip has two options. The first integrates a 10 GHz LC PLL, so that only a low-frequency reference enters the package. The second brings the clock in through ground–signal–ground probe pads with an on-chip 50Ω termination and a CML-to-CMOS converter; the pad, ESD, and termination capacitance must then keep the input return loss below −10 dB up to about 20 GHz, and the converter’s added jitter enters the output budget directly. Packaging follows from the same argument. A bond wire contributes roughly 1 nH per millimetre, about 63Ω of reactance per nanohenry at 10 GHz, which rules out a bond-wired, matched 10 GHz input; first silicon is therefore probed on-wafer, and a packaged part uses flip-chip assembly or the on-chip PLL. The package inductance still matters at low frequency because it sits in the supply loop that sets the dominant supply-induced jitter term in Section 3.4: the LDO and on-chip decoupling have to hold the 100μV budget with the package included. The output, at most 1 GHz, leaves through a 50Ω CML or LVDS driver. The calibration itself can be verified with standard instruments. Femtosecond edge placement is not observed directly but through the fractional spur level and the integrated jitter at the output, measured with a phase-noise analyzer—the quantities in Section 5.7—while the stored calibration codes are read out through a serial interface.

**Slow drift: temperature and aging (Class 3).** Temperature and aging form the slow-drift class of Table 1: fixed on the timescale of one calibration but varying over hours to years. The calibration is self-referenced — the calibration chain is nominally identical to the main chain and shares its process, supply, and temperature, so drift common to both largely cancels in the differential edge comparison, and only the residual differential drift degrades the stored calibration. It is also foreground: it runs at startup and freezes. A temperature sweep of both chains from −40 to 120 °C (Figure 17) quantifies what that costs. The common-mode delay drift is substantial, approximately 15 fs per degree, but it is also largely benign: a drift shared by every output edge is a static phase offset, not jitter, and matters only for multi-FOD skew. The operative metric is the differential drift between the main and calibration chains since it invalidates the stored calibration. Referenced to 27 °C, the differential tracking stays within the ±0.25 DTC LSB ($\pm \frac{1}{2}$ calibration LSB) budget across the entire −40 to 40 °C band, so a foreground calibration performed anywhere in that range survives ordinary ambient excursions without refresh. Above 40 °C the chains diverge at approximately 1.4 DTC LSB (2.8 calibration LSB) per 10 °C, which under a half-calibration LSB error budget implies a re-calibration roughly every 2 °C of die temperature change in the hot regime. The interval is gain-gated: it applies to the gain code, which sets the accuracy of the interpolation, while the offset code is re-aligned at every refresh anyway. A refresh of the offset and gain codes alone costs $44\;M_{0}\;\approx \;4.8\;\times \;10^{3}$ comparisons, or 4.8μs at N=10 (Section 4.6), so frequent hot-regime refresh is affordable without re-running the bit-weight extraction, whose ratio-based weights share, to first order, the common temperature coefficient of the ladder. Aging and slow supply drift remain to be characterized in silicon; the self-referenced structure is expected to suppress the common-mode part of each, and an on-chip temperature, aging, or supply monitor can trigger the same refresh.

**Division-ratio range.** The settled-accuracy budget (6), with R=50Ω and $C_{\mathrm{tot}}=2$ pF (τ=100 ps), sets the operating range of this design at N≥10 for a 10 GHz input, covering output frequencies up to 1 GHz. Smaller ratios require a proportionally smaller ladder time constant, and therefore more static reference current; ratios below that bound are outside the scope reported here.

**Detector limits.** The transistor-level runs use an ideal comparator; the physical element intended for it is the D flip-flop of the companion divider [22]. Section 3.5 separates the three ways a real comparator enters: a static offset cancels in every stored difference, random noise sets the repetition depth, and metastability is bounded by the resolution time of one divided period [24]. The system-level Monte Carlo in Section 5.7 confirms the first two quantitatively (Figure 16a,b): with the repetition depth scaled to the comparator noise, the post-calibration INL is unchanged up to 200 fs of input-referred comparator noise, at the cost of a longer calibration, and a static offset has no effect until, together with the chain mismatch, it pushes an alignment outside the fine-delay range. A transistor-level characterization of the flip-flop’s offset, noise, and kickback onto the timing nodes, inside the calibration loop, is the remaining validation step for this block.

**Scope of the sensor-facing claims.** The sensor system quantities in Section 1.1 are consequences of the timing results reported here, not separate experiments: the aperture-limited figures follow from (1) applied to the random jitter budget in Section 3.4 and the deterministic residue in Section 5.7. No sensor front end was built or co-simulated here, so these figures bound what the clock contributes to a readout, not what a complete readout achieves: in a physical time-of-flight channel the detector, the illumination source, and the time converter each add error terms that ordinarily dominate the femtosecond-scale contribution of the clock. Quantitatively, the single-shot precision of a pulsed-LiDAR channel combines laser pulse width, detector time jitter, and time converter error in quadrature, and is usually dominated by the laser pulse width, with repeated measurement and centroid extraction recovering precision beyond the converter’s own quantization [5]; correspondingly, the jitter of a sampling system decomposes into sampling circuit, input signal, and sampling clock terms, of which a clock generator such as the one presented here sets only the last [6]. The claim is correspondingly narrow: this work reduces the divider’s own contribution to the timing error budget of such systems and quantifies it, and the delay step itself is not a sensor resolution.

**Quantization-induced spurs.** Calibration linearizes the DTC but does not remove the hard quantization of the delta-sigma residue onto the 12-bit code grid; this resolution mismatch generates its own spur family independent of INL, with patterns set by the fractional word [23]. Input dithering of the quantizer, which is orthogonal to the calibration presented here, is the appropriate countermeasure and is compatible with the architecture.

## 7. Conclusions

A DAC-based constant-slope DTC for fractional output dividers was designed in 22 nm FDSOI and evaluated in simulation with a 10 GHz input clock. A timing scheme that assigns one input clock period to discharge and N−1 periods to DAC settling removes the settling obstacle that had excluded this architecture from high-speed FODs. A complete foreground calibration was derived: an exact gain-measurement condition requiring a two-calibration LSB code retreat, a geometrically convergent alternating offset/gain loop, a delay-domain radix weight extraction that measures the five mismatch-dominant bit weights by averaged carry-pair alignments inside a fixed 124-decision sequence, and a closed-form account of the smooth curvature that such per-bit calibration cannot observe and that the regulated cascode suppresses at the source. Transistor-level simulations show chain tracking of ρ=0.94 under full mismatch after sizing, an 8-bit fine-delay vernier dimensioned to the ±3σ chain-mismatch bound and offset–gain convergence below half a calibration LSB in two iterations. A 200-die system-level Monte Carlo anchored to those statistics shows the median peak INL falling from 4.1 to 0.32 LSB, with 199 of 200 dies below 1 LSB and the remaining die outside the fine-delay range; the worst fractional spur falling from −75.5 dBc to the −91 dBc quantization floor; and the deterministic jitter falling from 53 to 7.4 fs rms, with the accuracy maintained up to at least 200 fs rms of comparator noise when the repetition depth is scaled. Where existing FOD calibrations estimate the DTC INL statistically through LMS loops, the methodology presented here measures the bit weights deterministically, in a fixed number of SAR steps, with an analytic account of what remains; to the authors’ knowledge, it is the first transfer of data converter bit-weight self-calibration to the delay domain of an FOD. For sensor systems, the calibrated divider’s contribution to the aperture error of a sampled front end is 28 fs rms. Layout extraction and silicon validation, along the path in Section 6, are the next steps.

## Figures and Tables

**Figure 1:**
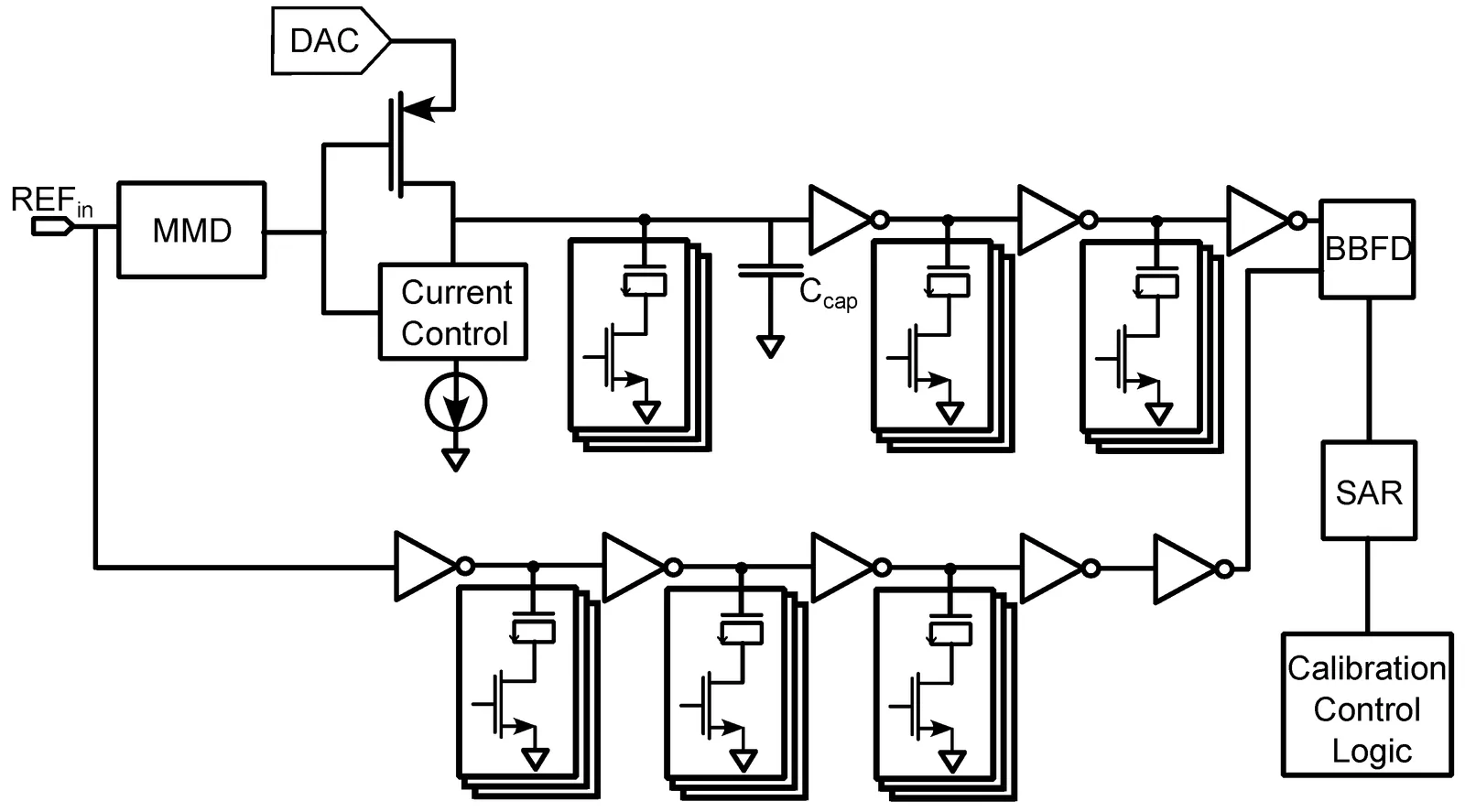
Architecture of the FOD with the proposed foreground calibration infrastructure. The input reference (${\mathrm{REF}}_{\mathrm{in}}$) drives the multi-modulus divider (MMD); the R-2R DAC and the current-control block program the constant-slope delay cell, and the main signal path (top) is a cascade of such cells with per-node timing capacitance $C_{\mathrm{cap}}$. A nominally identical calibration chain (bottom) is compared against the main path by a bang-bang phase detector (BBFD), modeled as an ideal comparator (Section 3.5); the SAR engine with its calibration control logic sets the fine-delay, gain, and per-bit trim controls. The delta-sigma modulator that steers the division ratio and supplies the quantization residue $\varepsilon_{q}\left[k\right]$ to the DAC (Section 2) is omitted for clarity.

**Figure 2:**
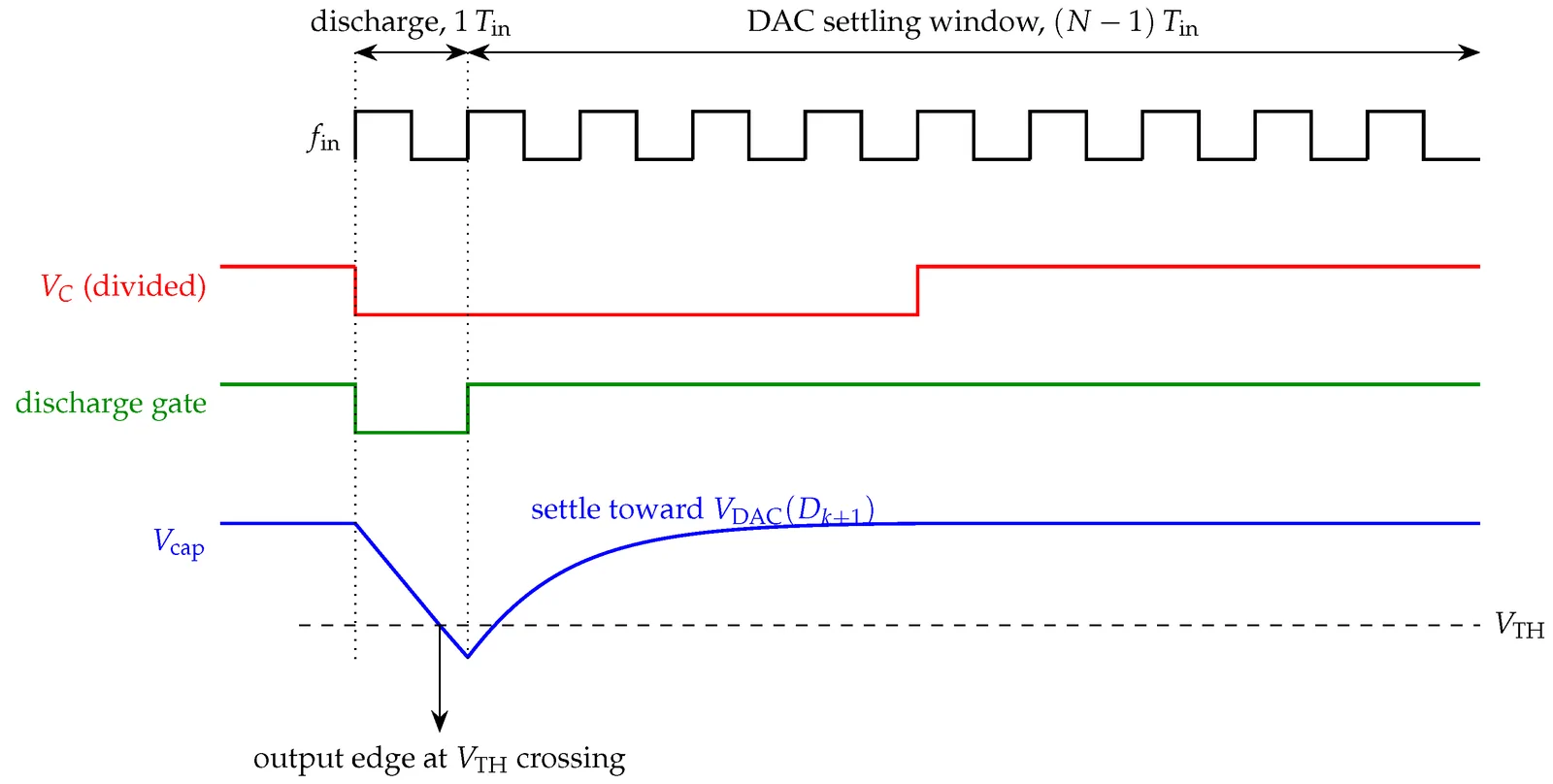
Timing allocation for N=10, the minimum division ratio in Section 2. The falling edge of the divided clock $V_{C}$ opens the discharge gate for exactly one input clock period; the constant-current ramp crosses the inverter threshold $V_{\mathrm{TH}}$ within this window and produces the output edge. For the remaining N−1 input periods the DAC recharges the timing node toward the next code voltage, so the settling window grows with the division ratio as in Equation (6).

**Figure 3:**
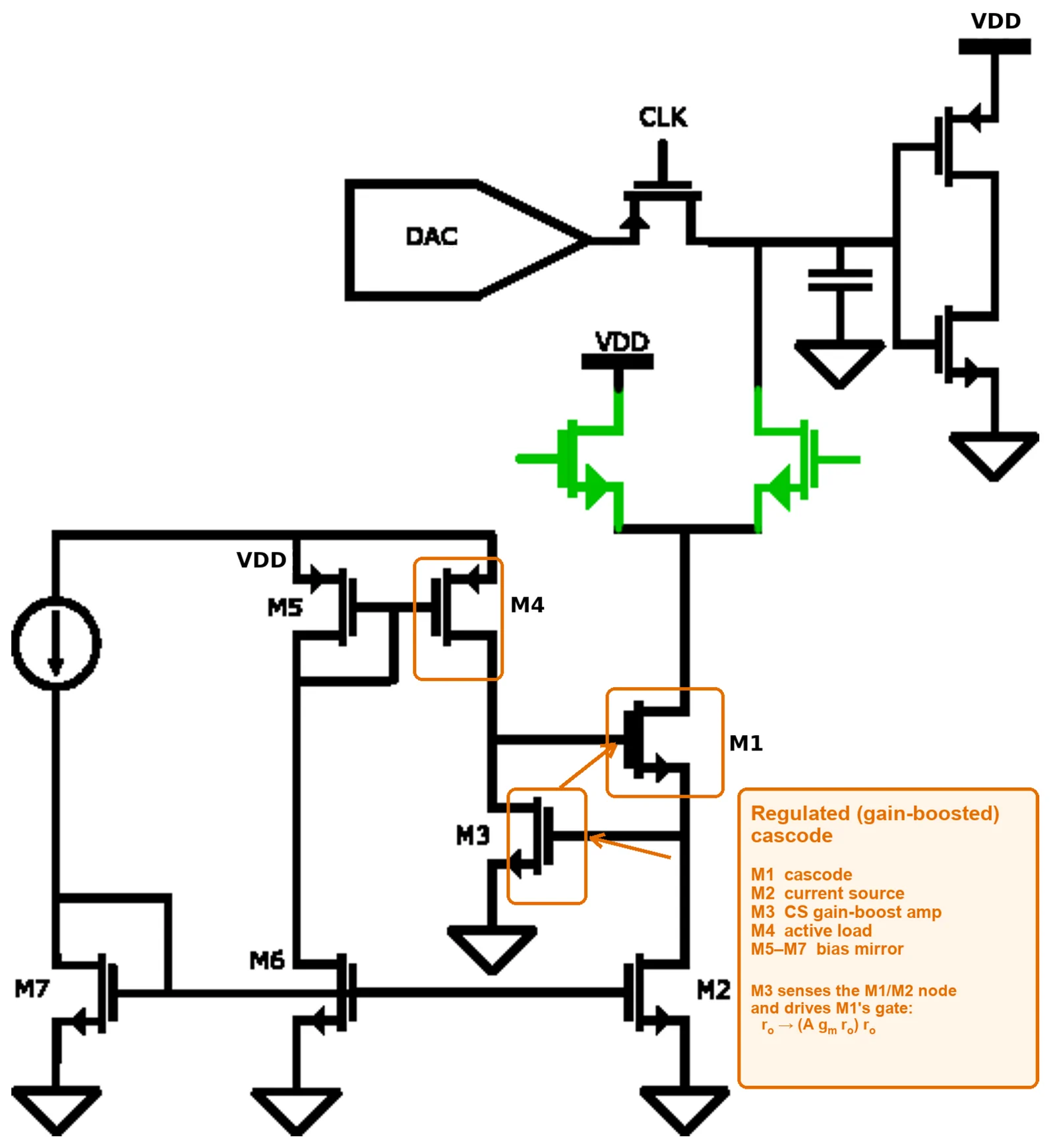
Schematic of the constant-slope delay cell. The R-2R DAC drives the timing node through the clock-gated precharge switch; the node capacitance is the parallel combination of a fixed capacitor and the varactor formed by the threshold-inverter input. The discharge current is provided by the cascode stack M1–M2, regulated by a common-source gain-boost amplifier (M3 with active load M4) in the feedback loop that senses the M1–M2 cascode node and drives the gate of M1, raising the output resistance $r_{o}$ and suppressing the curvature bow (Section 4.7); the M5–M7 mirror sets the bias current, and the cross-coupled pair (green) sharpens the handover between the charge and discharge phases.

**Figure 4:**
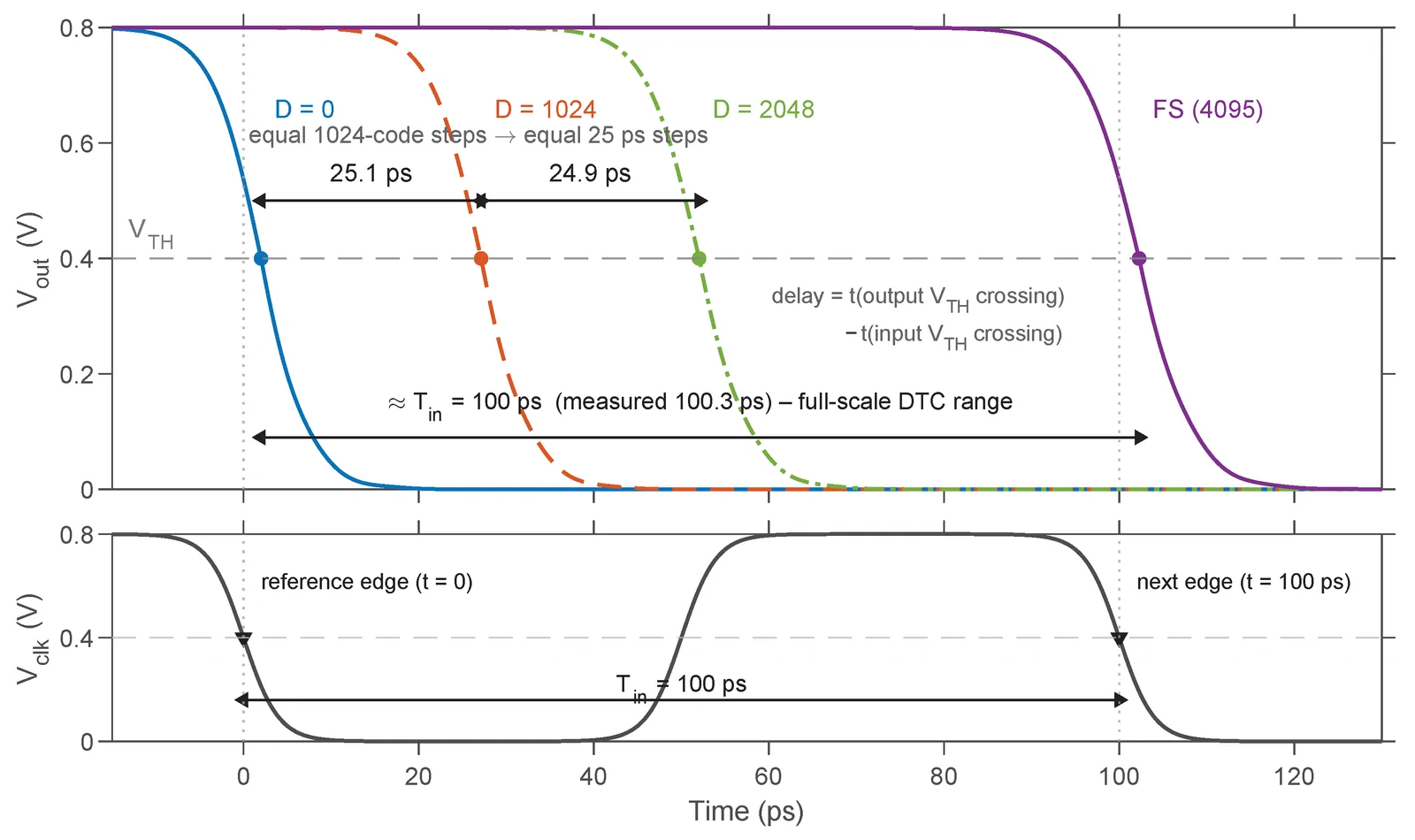
Post-schematic transient of the delay cell output for DAC codes 0, 1024, 2048, and full scale (**top**) with the 10 GHz-class input clock (**bottom**). Both output transitions displace monotonically with the code; the delay is measured between threshold crossings of the input and output falling edges as defined in Section 3. The delay is referenced to the code-0 edge, so the common code-independent propagation offset $t_{0}^{\prime }$ of Equation (8) is suppressed; the cell is shown uncalibrated, so the measured full-scale span (100.3 ps) is near, not exactly, the ideal one-LSB-short range $\left(2^{12}-1\right)/2^{12}\;T_{\mathrm{in}}\approx 99.98$ ps of Equation (3).

**Figure 5:**
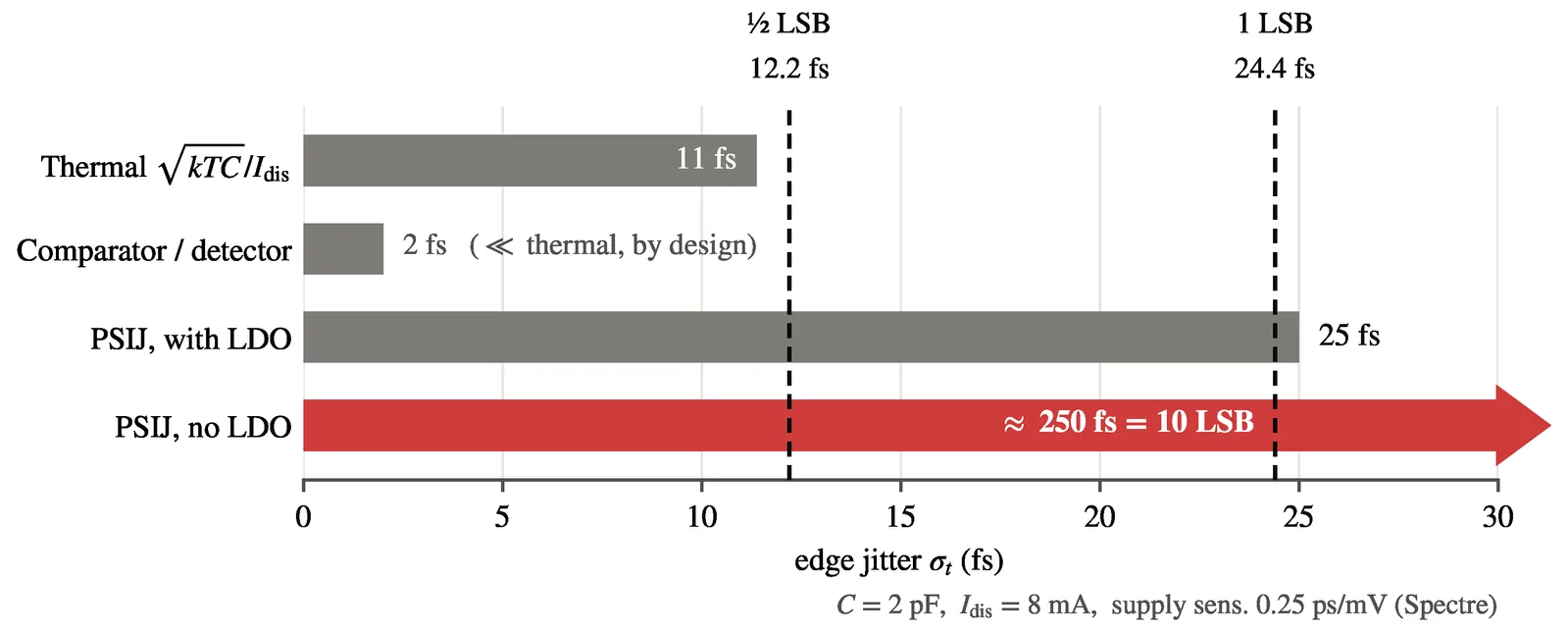
Edge-jitter budget at the design point (C=2 pF, $I_{\mathrm{dis}}=8$ mA). Thermal noise sits below the half-LSB line and comparator noise is designed well below the thermal floor; power supply-induced jitter dominates, reaching ten LSBs unregulated and requiring a dedicated LDO ($\sigma_{V_{\mathrm{DD}}}≲100\;\mu$V) to bring it to one LSB.

**Figure 8:**
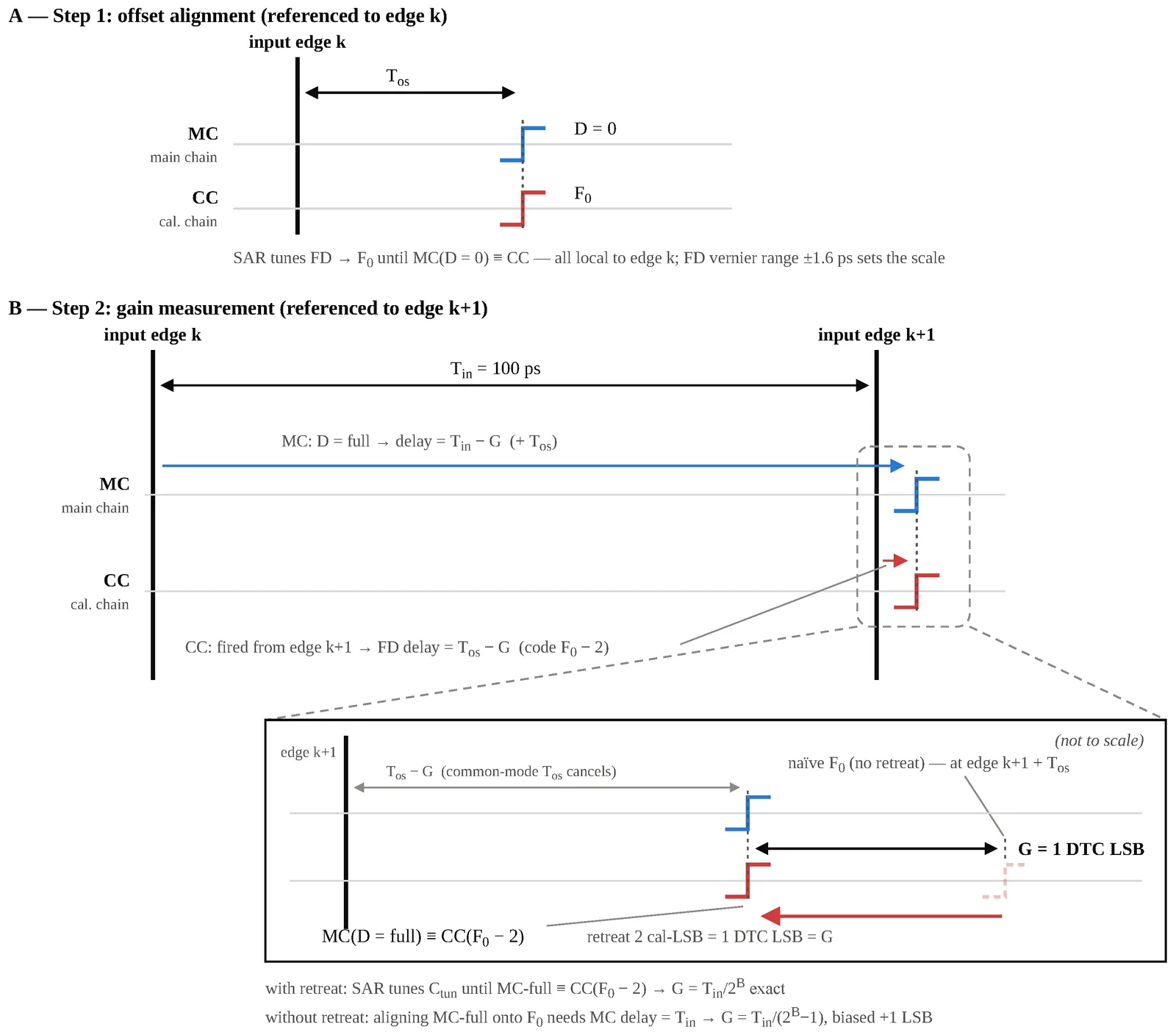
Gain measurement with the two-LSB correction; the fine-delay (FD) element delays the calibration-chain edge, so every CC delay is positive. (**A**) Step 1, offset alignment referenced to edge *k*: the SAR tunes the FD to $F_{0}$ until the main-chain (MC) edge at D=0 coincides with the calibration-chain (CC) edge, both sitting a common offset $T_{\mathrm{os}}$ after edge *k*. (**B**) Step 2, gain measurement referenced to edge k+1: the MC full-code edge, fired from edge *k*, lands $T_{\mathrm{os}}-G$ after edge k+1 because its delay $\left(2^{B}-1\right)\;G=T_{\mathrm{in}}-G$ is one DTC LSB short of a full period, while the CC edge is fired from edge k+1 with a small FD delay. Retreating the FD by two calibration LSBs (one DTC LSB, =G) moves the CC edge earlier by exactly *G* to coincide with the MC edge—the common $T_{\mathrm{os}}$ cancels in the comparison—so tuning $C_{\mathrm{tun}}$ converges to the exact gain $G=T_{\mathrm{in}}/2^{B}$ of Equation (18); without the retreat, aligning onto $F_{0}$ would force $G=T_{\mathrm{in}}/\left(2^{B}-1\right)$, biased high by one LSB.

**Figure 9:**
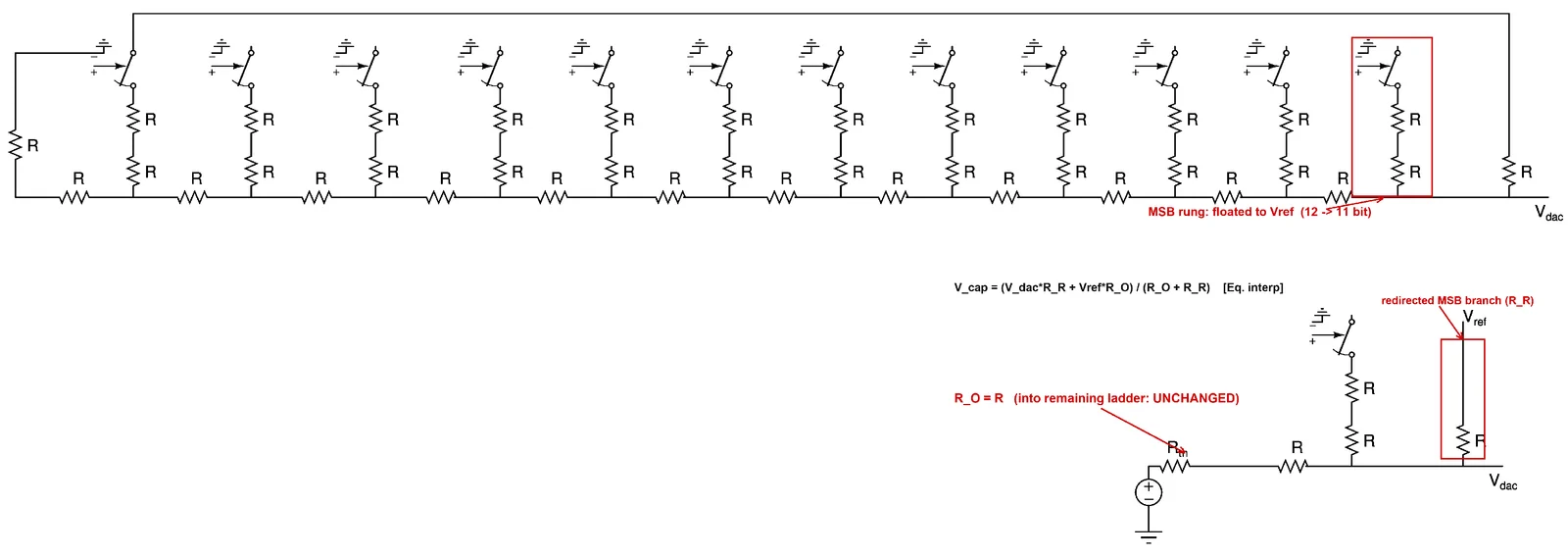
R-2R reconfiguration used for radix weight extraction, drawn for the step that converts the 12-bit ladder into an 11-bit ladder. (**Top:**) the full 12-bit ladder; the MSB branch (red box) is disconnected from the code and tied to $V_{\mathrm{ref}}$, dropping the ladder to 11 bits. (**Bottom-right:**) the resulting Thévenin/interpolation equivalent, with the redirected MSB branch shown as $R_{R}$ (red); the output is the interpolation of Equation (21) between the remaining ladder and the reference, while the Thévenin resistance seen by the timing capacitor, $R_{O}\equiv R_{\mathrm{th}}$, is unchanged (=R), so the settling dynamics are preserved. Repeating the step bit by bit exposes each weight as a measurable displacement of the output edge around the mid-scale alignment.

**Figure 10:**
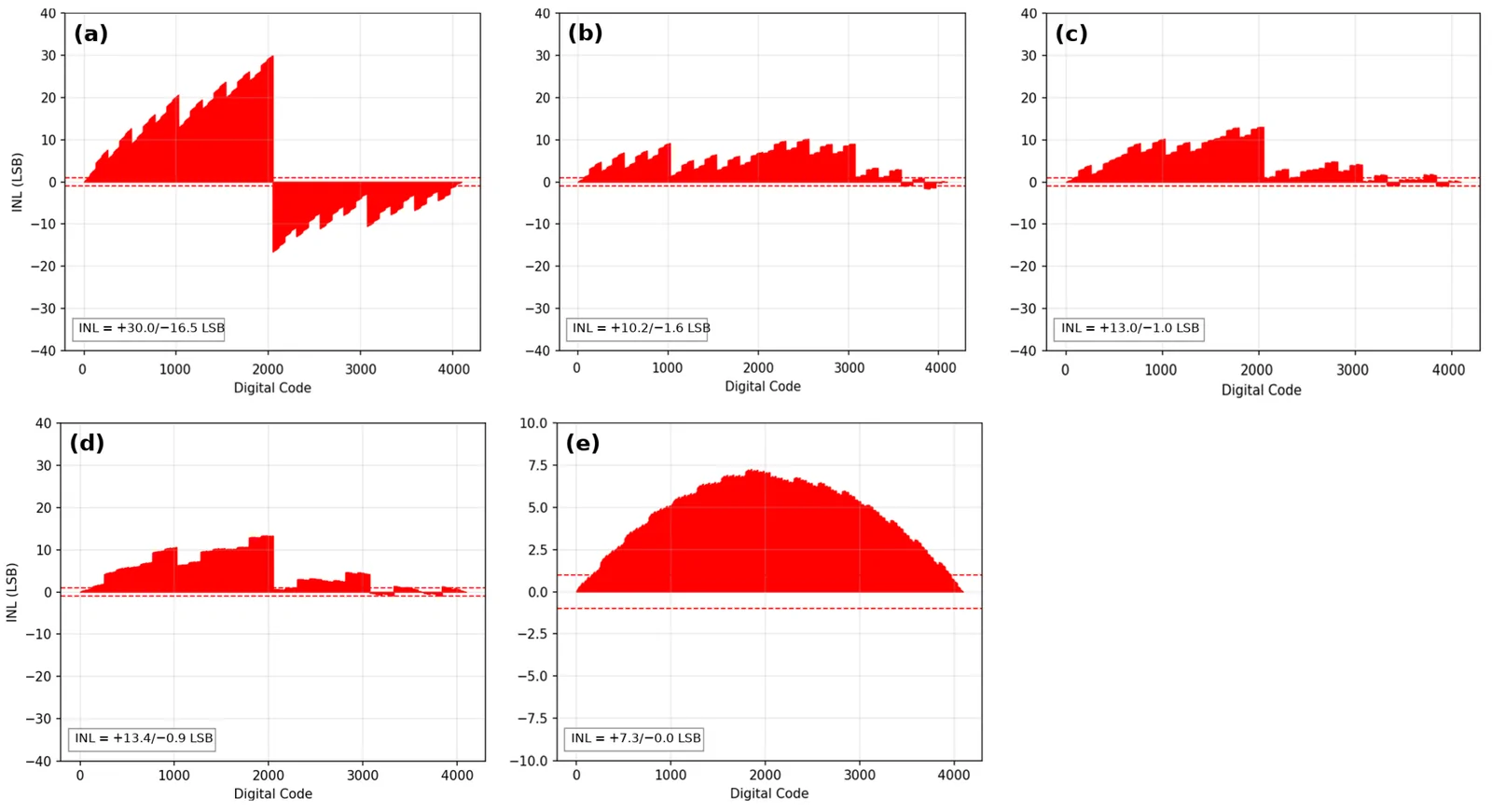
Radix weight-extraction calibration applied step by step to a gain-calibrated 12-bit DTC (transistor-level simulation in Cadence Spectre 21.1, Virtuoso IC6.1.8, Cadence Design Systems, Inc., San Jose, CA, USA; calibration-chain trim resolution 0.5 LSB), for a deliberately high-mismatch cell—an injected worst case outside the ±13 LSB envelope of the 200 Monte Carlo samples, chosen so that every trim step is visible. Gain calibration corrects the slope but leaves the bit-weight nonlinearity untouched. (**a**) Step 0, the gain-calibrated starting point: +30.0/−16.5 LSB bit-weight INL. (**b**) Step 1, after trimming the MSB $b_{11}$: +10.2/−1.6 LSB. (**c**) Steps 2–3, after trimming the next major carry pair $b_{10},b_{9}$: +13.0/−1.0 LSB. (**d**) Steps 4–12, the full MSB-to-LSB pass evaluated against a stale calibration reference: +13.4/−0.9 LSB, which still carries major carry structure. (**e**) the same pass re-run in LSB-to-MSB order, which removes the stale reference residual: the major carry structure disappears and only the +7.3/−0.0 LSB quadratic curvature bow remains, the limit per-bit trimming cannot reach, consistent with (29)–(30). Note the change of vertical scale in (**e**). At the design averaging depth $M_{0}=108$ (Section 4.6), the measurement noise of the calibration is ≈1.5 fs (0.06 LSB), below the resolution of the plot. Red dashed lines: ±1 LSB.

**Figure 11:**
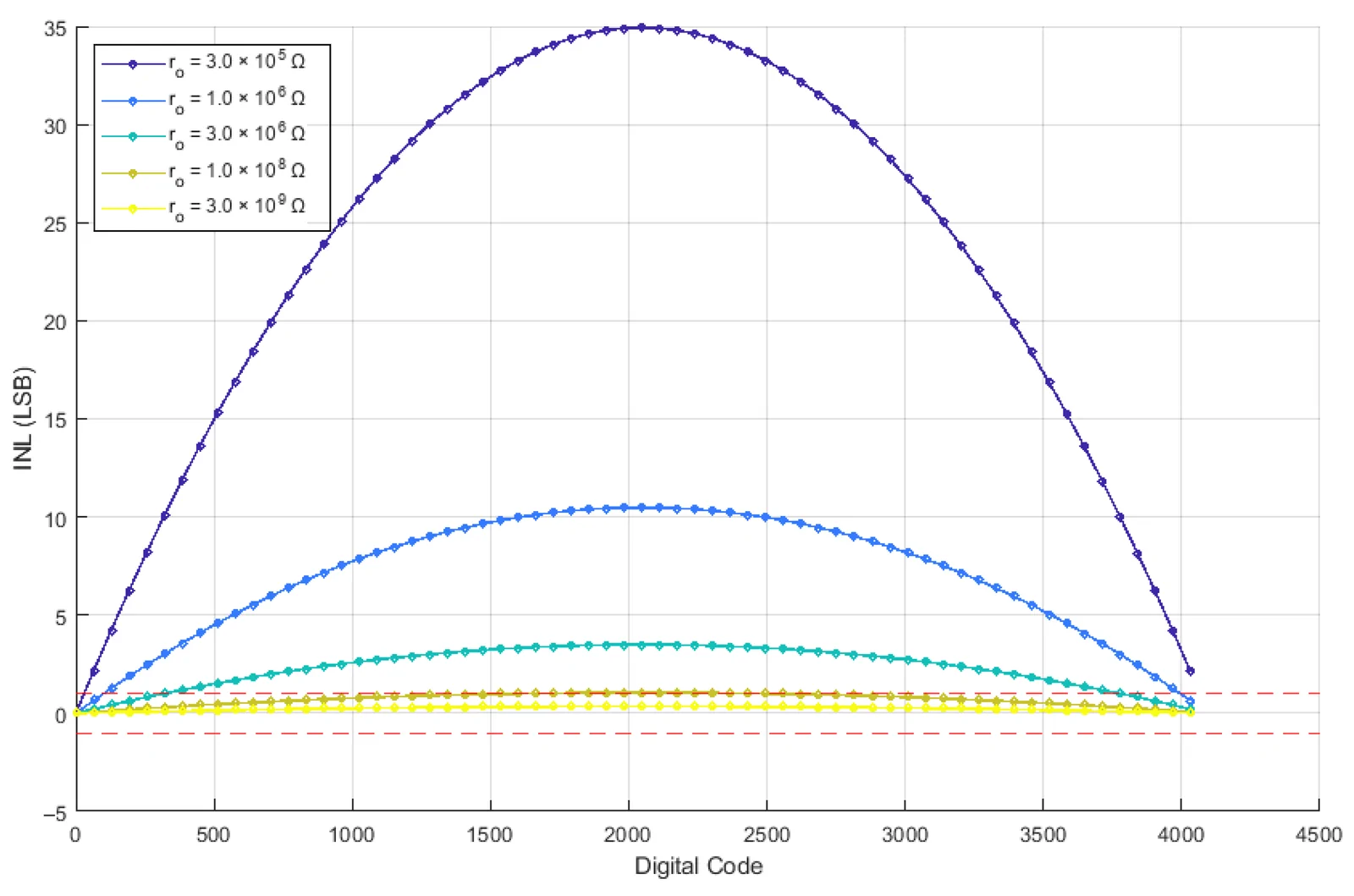
Post-calibration residual INL versus current-source output resistance $r_{o}$, on the characterization testbench (transistor-level Spectre, post-processed in MATLAB R2024b, The MathWorks, Inc., Natick, MA, USA); the $1/r_{o}$ scaling, not the absolute scale, is what transfers to the design point, where Equation (31) applies. Simulated peak INL follows the $1/r_{o}$ law of (30) for $r_{o}\leq 3\times 10^{6}\;\Omega$ and saturates at a residual floor of order one LSB beyond, dropping below one LSB for $r_{o}≳10^{8}\;\Omega$. As in Figure 10, the calibration measurement noise at the design depth $M_{0}=108$ (≈1.5 fs) sits well below the plotted curvature. Dashed lines: +1 LSB (red) and −1 LSB (gray).

**Figure 12:**
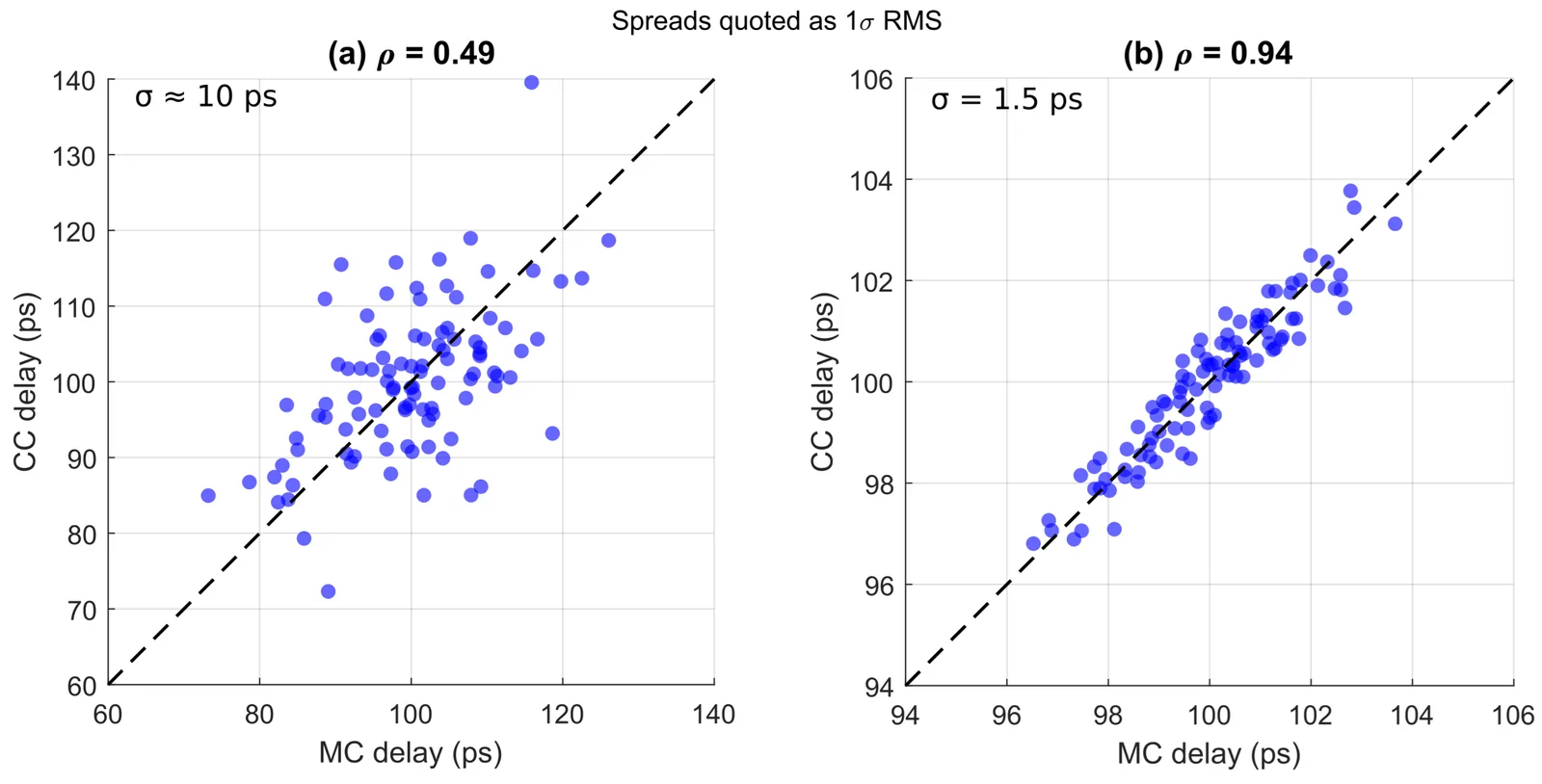
Main-chain versus calibration-chain delay over 100 Monte Carlo samples from a single transistor-level Spectre run (both chains in one netlist), with local random mismatch enabled; spreads are quoted as 1σ RMS. (**a**) Initial sizing: the main-chain spread is ≈10 ps RMS (a ±30 ps, ±3σ, peak bound) and the correlation collapses to ρ=0.49, which no fine-delay range could economically cover. (**b**) After enlarging the DAC unit-resistor area by 32× and the delay element by 64× (both at fixed nominal *R* and delay): the spread contracts to 1.5 ps RMS, a 6.7× reduction consistent with the $\sqrt{32}$–$\sqrt{64}$ Pelgrom area scaling, and the correlation recovers to ρ=0.94.

**Figure 13:**
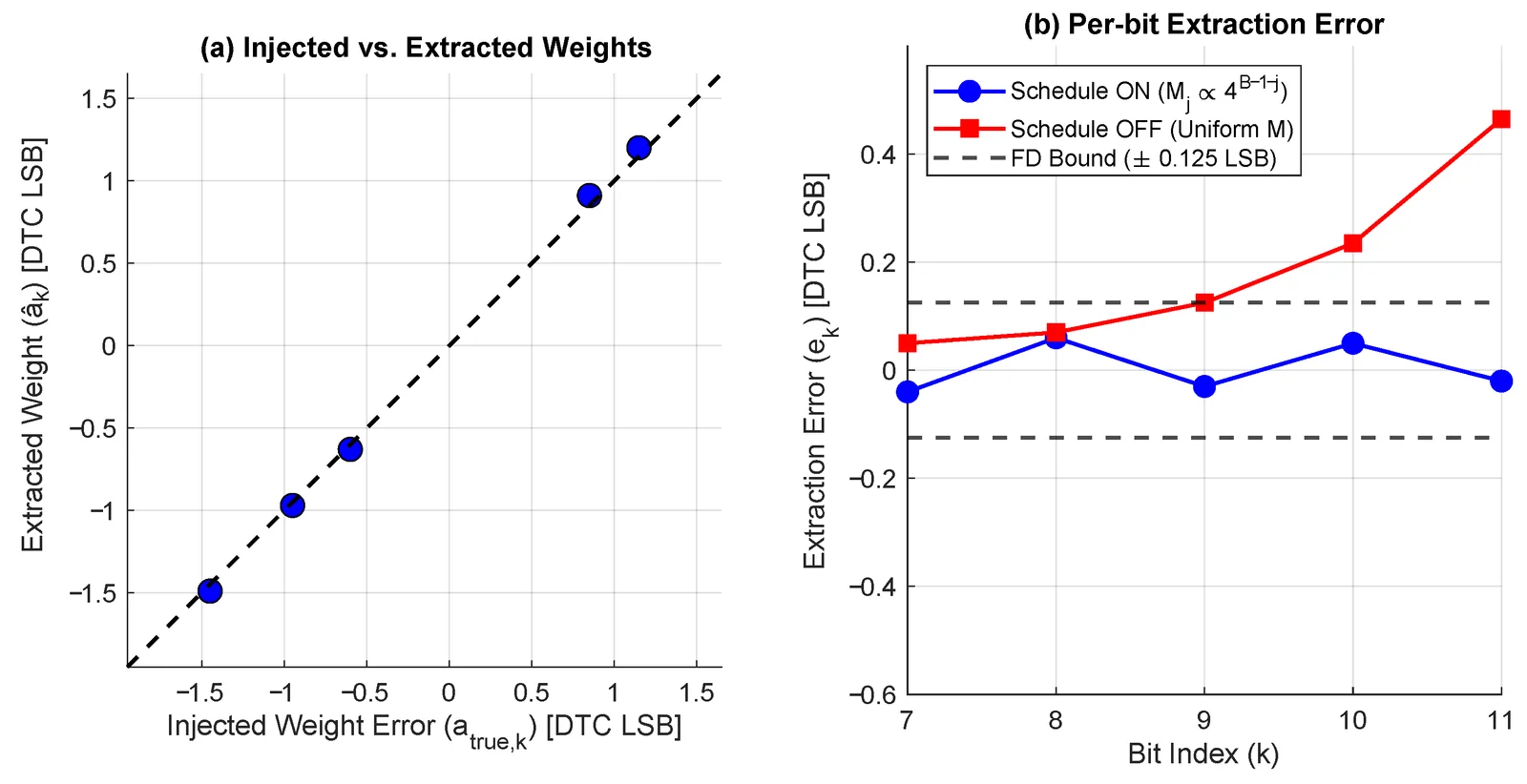
Closed-loop radix weight extraction, representative transistor-level run. (**a**) The five calibrated MSB weight errors extracted by the SAR carry-pair procedure and the triangular solve of Equation (25) fall on the injected values (dashed identity line). (**b**) Per-bit extraction error: with the repetition schedule $M_{j}\propto 4^{(B-1)-j}$ active (blue) the error stays inside the ±0.125 DTC LSB band (one quarter of the vernier step, dashed) with no growth across bit index, whereas uniform repetition (red) lets the error grow toward the MSB with the amplitude ratios predicted by Equation (26)—confirming both the noise propagation law and the necessity of the schedule.

**Figure 14:**
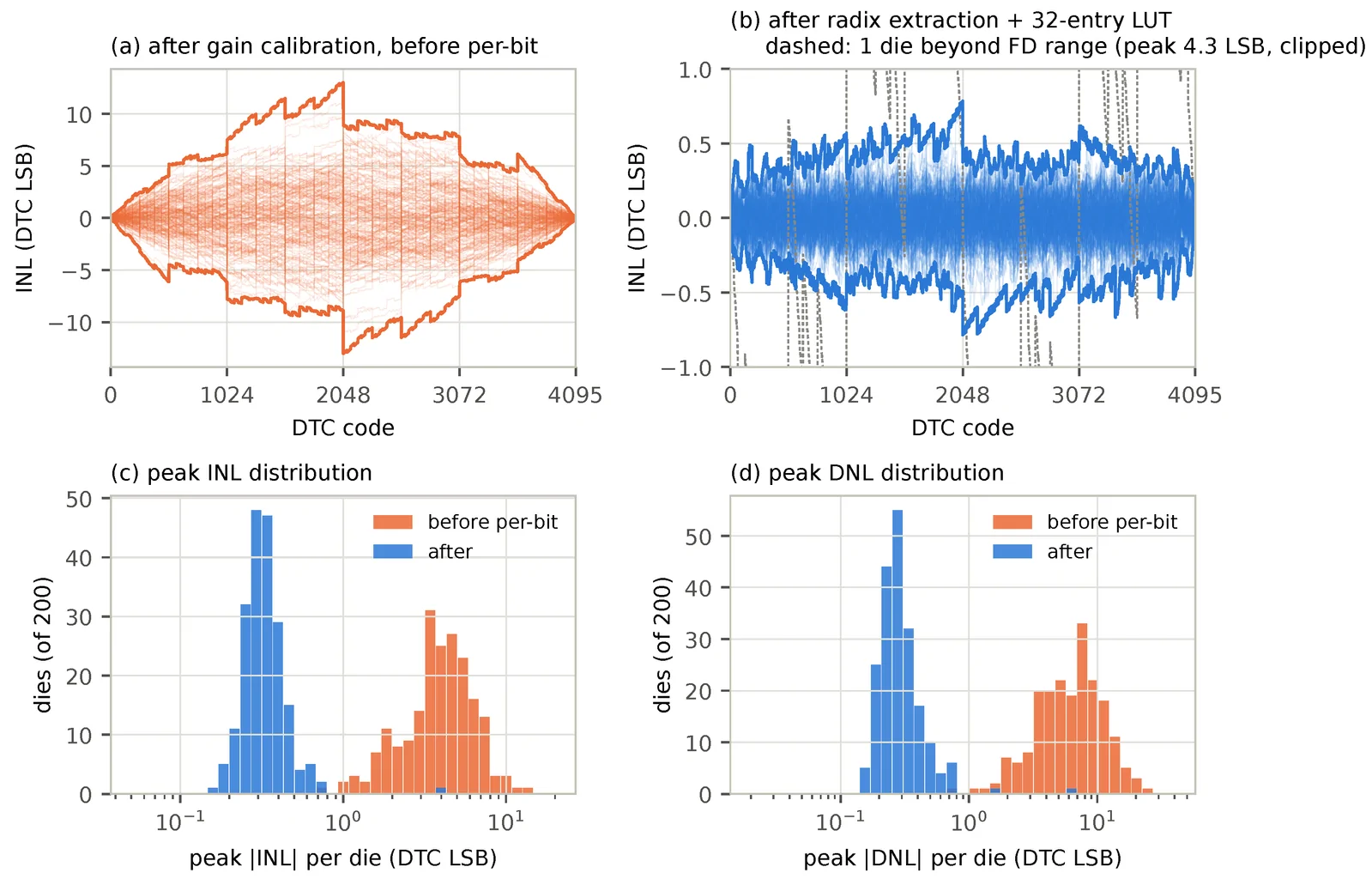
System-level Monte Carlo over 200 dies; unit-resistor ratio mismatch 0.42%, set to reproduce the ±13 LSB transistor-level envelope. (**a**) Endpoint-fit INL after gain calibration, before the per-bit correction; bold lines are the envelope. (**b**) After radix extraction and the 32-entry look-up table, on a different vertical scale; the one die whose chain-to-chain offset exceeds the fine-delay range (dashed) is clipped. (**c**) Peak INL and (**d**) peak DNL per die, before and after. The smooth V-to-t curvature is excluded; it adds one-for-one (Section 4.7).

**Figure 15:**
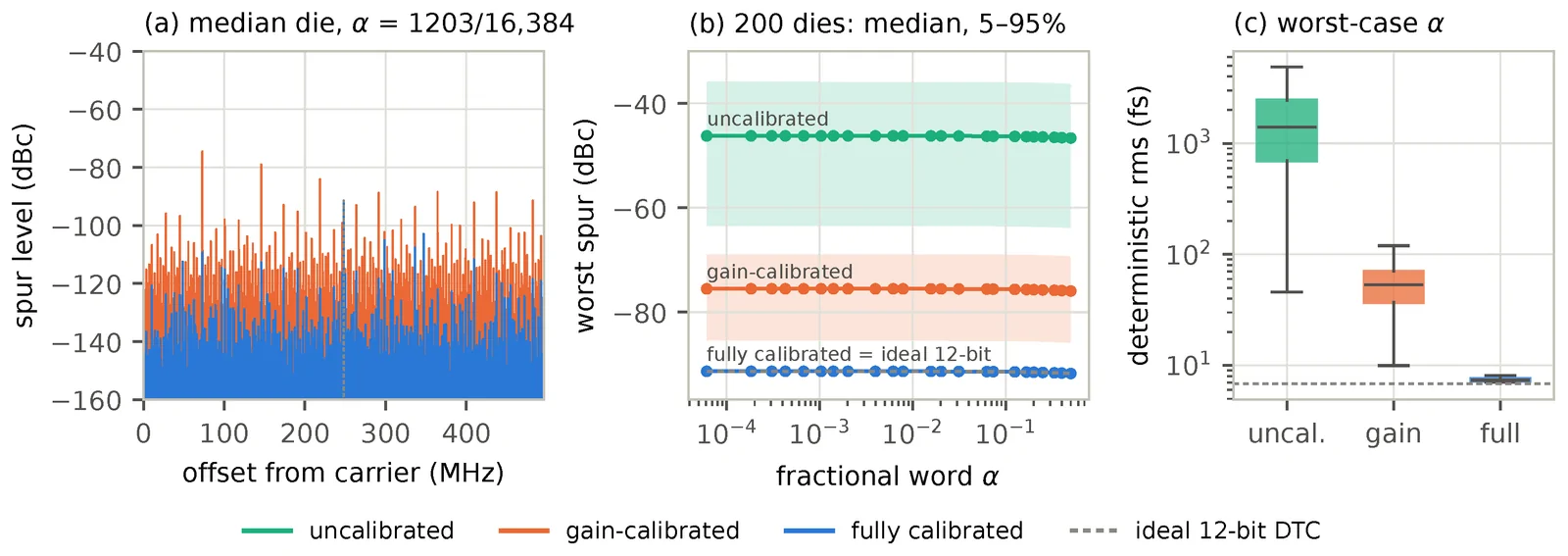
FOD output for the 200 dies of Figure 14: 10 GHz input, N=10, first-order delta-sigma modulator, 16,384 output edges per fractional word. (**a**) Phase spectrum of the median die at α=1203/16384. (**b**) Worst spur versus fractional word, median and 5–95% band over dies; the fully calibrated dies coincide with the ideal 12-bit DTC, whose remaining tone is quantization-induced. (**c**) Deterministic rms edge-time error at the worst fractional word; dashed line, ideal 12-bit DTC. System-level simulation; random jitter excluded.

**Figure 16:**
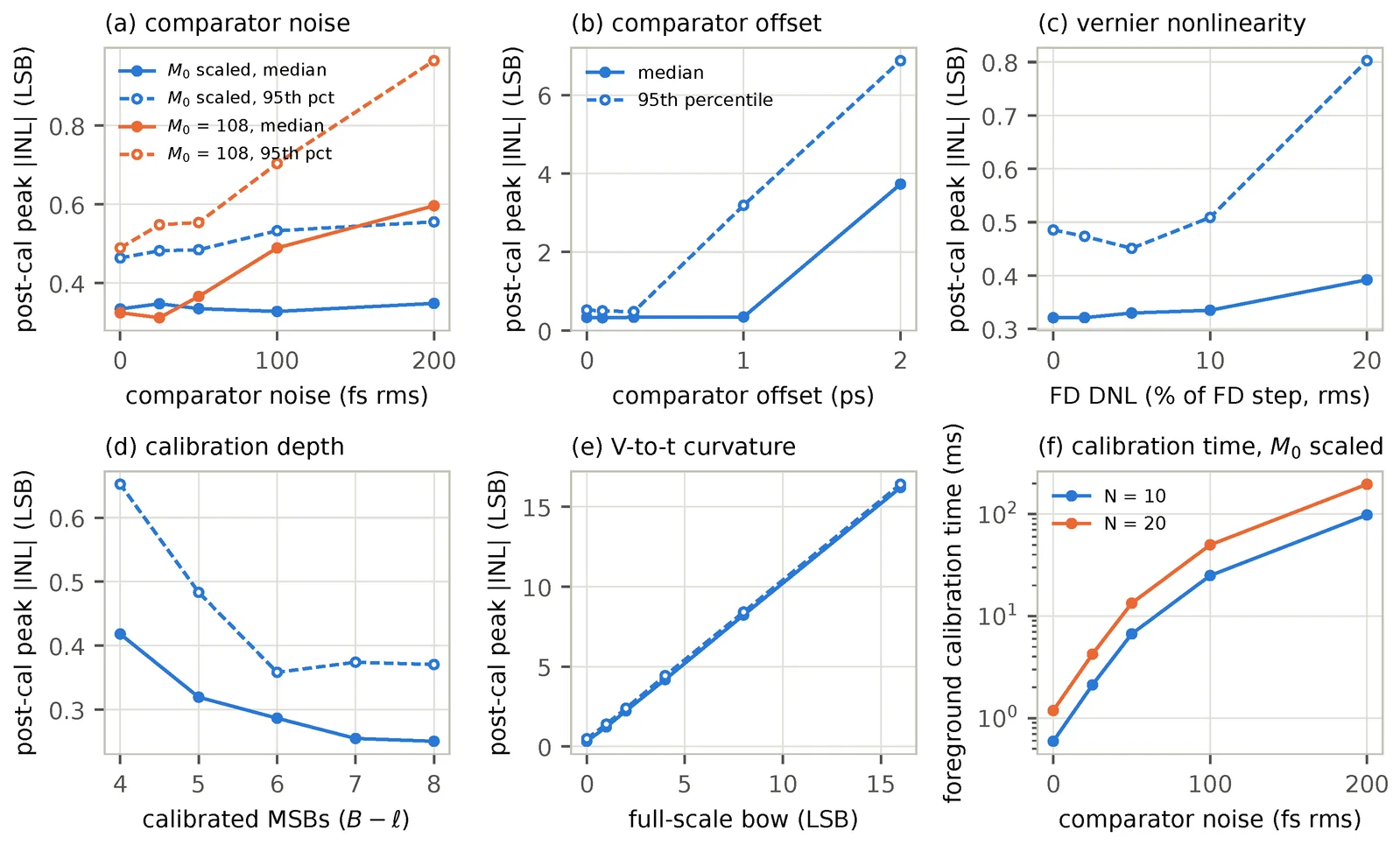
Sensitivity of the calibration to its measurement hardware, 60 dies per point; median (solid) and 95th percentile (dashed) of the post-calibration peak INL. Dies whose alignment leaves the fine-delay range are counted separately, except in (**b**), where that is the effect being swept. (**a**) Comparator input-referred noise, with the repetition depth $M_{0}$ scaled to the total noise (blue) or held at 108 (orange). (**b**) Static comparator offset. (**c**) Fine-delay vernier DNL. (**d**) Number of calibrated MSBs; this design calibrates five. (**e**) Full-scale V-to-t bow, which the calibration cannot observe. (**f**) Foreground calibration time with $M_{0}$ scaled, for N=10 and 20.

**Figure 17:**
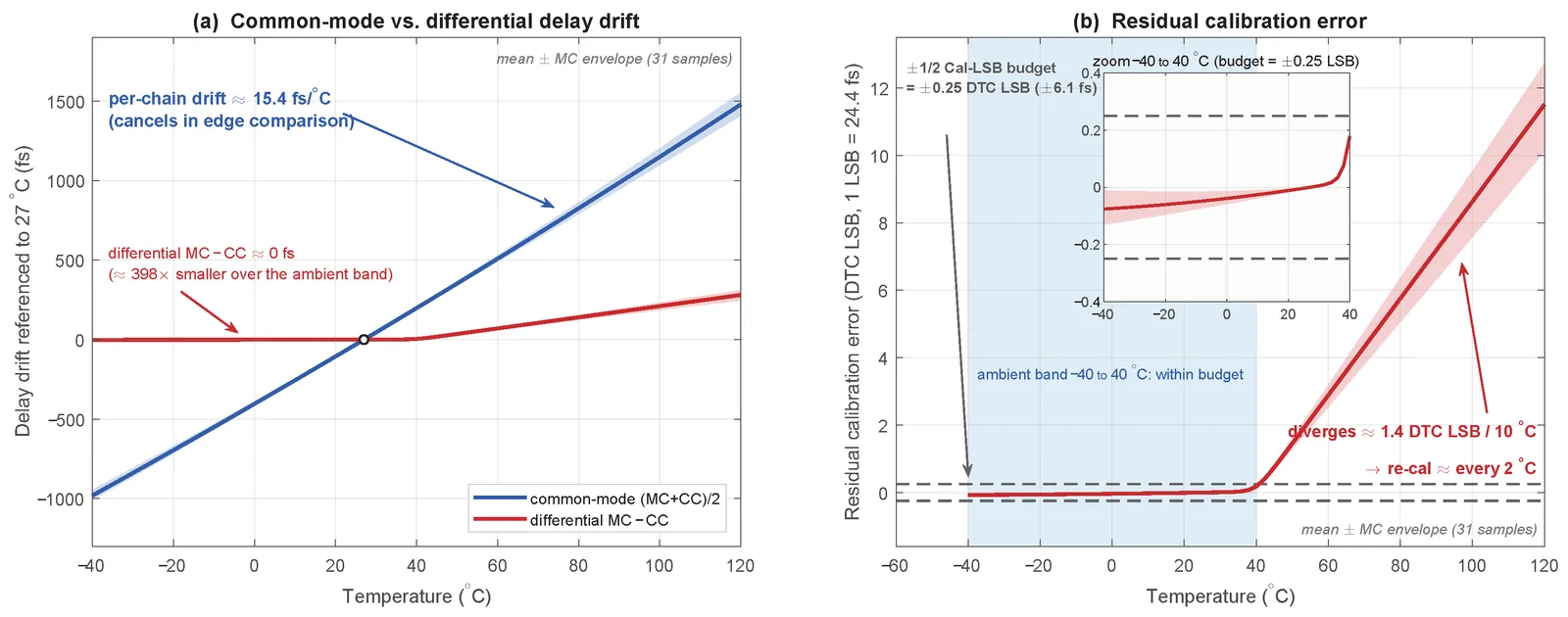
Temperature drift of the self-referenced calibration (Class 3 of Table 1); 31-sample Monte Carlo, both chains swept −40 to 120 °C and referenced to 27 °C. (**a**) Each chain drifts at ≈15 fs/°C (common-mode, blue), but this shared drift cancels in the differential edge comparison; the operative differential drift (red, MC−CC) is far smaller. (**b**) Residual calibration error: the differential stays within the ±0.25 DTC LSB ($\pm \frac{1}{2}$ calibration LSB, ±6.1 fs) budget across the −40 to 40 °C ambient band (inset) and diverges above 40 °C at ≈1.4 DTC LSB per 10 °C, setting a hot-regime re-calibration interval of ≈2 °C.

**Table 1:** The four classes of error source in the constant-slope delay cell and the distinct treatment each requires. On-chip measure-and-calibrate of the mismatch class (Class 2) is the central contribution of this work.

Class	Nature	Example in This Design	Treatment (Section)
1	real-time random noise	thermal (kT/C); supply-induced jitter	resource trade-off: size *C*, dedicated LDO (Section 3.4)
2	random, fixed at fabrication	R-2R bit-weight mismatch	measure and calibrate: on-chip radix weight extraction (Section 4.5)
3	fixed but slowly drifting	temperature, aging	self-referenced differential measurement; periodic refresh of the offset and gain codes (Section 6)
4	measurable and physically modeled	finite current-source output resistance $r_{o}$	circuit innovation: regulated cascode suppresses the source (Section 4.7)

**Table 2:** DAC options for generating $V_{\mathrm{start}}\left(D\right)$ in a constant-slope DTC, compared on the properties that matter for delay-domain bit-weight extraction.

	C-DAC [13,14]	R-String	Current Steering	R-2R (This Work)
Static INL set by	capacitor ratio matching	$2^{B}$ tap ratios	unit-current matching, output impedance	resistor ratio matching
Output resistance vs. code	charge sharing couples all bits	tap dependent	code dependent (finite $r_{o}$ of active cells)	constant, $R_{\mathrm{th}}=R$
Settling vs. code	code dependent	code dependent	code dependent	code independent
Bit weights accessible for extraction	no; redistribution couples all bits	no binary weights ($2^{B}$ unknowns)	yes, but the output load adds its own error	yes, by reconfiguration at unchanged dynamics
Main cost	kT/C, switch charge injection	$2^{B}=4096$ units	static current, output impedance	static reference current, switch $R_{\mathrm{on}}$

**Table 4:** Peak post-calibration INL versus current-source output resistance $r_{o}$ on the characterization testbench (Figure 11).

$r_{o}$ (Ω)	Peak INL (LSB)	$\mathrm{INL}\cdot r_{o}$
$3.0\times 10^{5}$	35.0	$1.05\times 10^{7}$
$1.0\times 10^{6}$	10.5	$1.05\times 10^{7}$
$3.0\times 10^{6}$	3.5	$1.05\times 10^{7}$
$1.0\times 10^{8}$	∼1.0	floor
$3.0\times 10^{9}$	<1.0	floor

**Table 5:** Transistor-level simulation setup.

Item	Setting
Simulator	Cadence Spectre 21.1 in Virtuoso IC6.1.8
Process	GlobalFoundries 22FDX PDK, version PDK_240621_155221_905; flipped-well devices in the delay cells
Supply, temperature	0.8 V; 27 °C nominal; −40 to 120 °C for the drift study (31 samples); −25 to 125 °C in seven steps for the offset–gain convergence study (200 samples)
Input clock	ideal 10 GHz pulse source ($T_{\mathrm{in}}=100$ ps), rise/fall time 12 ps, no jitter
Monte Carlo	process and local mismatch; process-only runs use the no-mismatch library variant (Section 5.2); 100 samples for chain tracking
Transient accuracy	errpreset conservative; reltol $10^{-3}$
Delay definition	time between the threshold crossings (350 mV) of the input and output falling edges, from the simulator’s crossing measurement; the code-independent offset $t_{0}^{\prime }$ is referenced out (Figure 4)
Comparator, SAR, look-up table	ideal Verilog-A models
System-level results	system-level Monte Carlo, Section 5.7; script and data in the Supplementary Materials

**Table 6:** Main-chain to calibration-chain delay correlation, 100 Monte Carlo samples, 22FDX.

Condition	Correlation ρ	Verdict
Process variation only (no-mismatch library)	0.994	tracking excellent
Process + local random mismatch, initial sizing	0.49	unusable
Process + local mismatch, resistor area ×32, delay element ×64	0.94	acceptable

**Table 7:** Comparison of FOD DTC calibration approaches: calibration mechanism (upper part) and reported performance (lower part).

	[3]	[2]	[1]	[11]	This Work
Technology	90 nm	28 nm	28 nm	28 nm	22 nm FDSOI
DTC type	delay line	delay line	delay line	delay line	constant slope
Calibration	background	background	background	foreground	foreground
Error detection	endpoint sampling	aux-PLL filter	split-DTC mutual ref.	mDTC mutual ref.	replica chain + SAR
INL correction	none	0th/1st/2nd-order LUT	sign-LMS codes	sign-LMS codes	per-bit radix weights
INL identification	—	statistical	statistical	statistical	deterministic
Convergence time	input dependent	input dependent	input dependent	input dependent	fixed step count
Residual bound	—	—	—	—	closed form, Equation (30)
Output frequency	0.625–200 MHz	10–300 MHz	123.46 MHz ^a^	123.46 MHz ^a^	≤1 GHz (N≥10, 10 GHz input)
DTC INL after cal.	n/r	n/r	n/r	n/r	0.32 LSB median, 0.54 p95 ^b^
Worst spur	−65 dBc	<−80 dBc	−81 dBc	−70 dBc	−91.3 dBc median, 12-bit quantization floor ^b^
Jitter	300 fs rms	310 fs rms (10 kHz–20 MHz)	191 fs rms	512 fs rms (10 kHz–20 MHz)	28 fs rms per edge (7.4 fs deterministic), DTC only ^b^
Area	0.008 mm^2^	0.084 mm^2^	0.024 mm^2^	0.01 mm^2^	not laid out
Power	1.5 mW	n/r	3.36 mW	3.6 mW	≈0.64 mW delay-cell core + 6.4 mW ladder reference ^c^
Calibration time	background	background	background	n/r	0.59 ms at N=10
Validation	measured	measured	measured	measured	transistor-level sim. (DTC), system-level sim. (FOD)

^a^ Frequency of the reported spur and jitter measurement. ^b^ System-level Monte Carlo over 200 dies, Section 5.7; excludes the smooth V-to-t curvature, which adds one-for-one (Section 4.7). ^c^ First-order estimates at $f_{\mathrm{out}}=1$ GHz: $I_{\mathrm{dis}}T_{\mathrm{in}}f_{\mathrm{out}}V_{\mathrm{DD}}$ for the cell, $V_{\mathrm{ref}}V_{\mathrm{DD}}/R$ for the ladder reference, Section 5.8.

**Table 8:** Comparison with constant-slope DTCs. Values for the prior works as stated in their abstracts.

	[12]	[13]	[14]	[15]	This Work
Technology	65 nm	28 nm	40 nm	28 nm	22 nm FDSOI
Start-voltage DAC	10-bit	9-bit C-DAC	10-bit C-DAC	current steering	12-bit R-2R
Resolution	19–185 fs	148 fs	563 fs	0.24 ps	24.4 fs
Range	19–189 ps	n/r	n/r	123 ps	100 ps
Clock rate	55 MHz	40 MHz	50 MHz	250 MHz	10 GHz input
INL	0.17% FS	1.07 LSB	0.96 LSB	53 fs (0.04% FS)	0.32 LSB (0.008% FS) median, calibrated
DNL	n/r	0.96 LSB	0.14 LSB	n/r	0.60 LSB p95, calibrated
Power	1.8 mW	31 μW	n/r	2.1 mW	≈0.64 mW
Calibration	none	none	none	none	deterministic foreground
Validation	measured	measured	measured	simulated	simulated

## Data Availability

The system-level Monte Carlo script in Section 5.7 and its outputs are provided as Supplementary Materials. The transistor-level simulation data are available on request from the corresponding author.

## Supplementary Materials

The following supporting information can be downloaded at https://www.mdpi.com/article/10.3390/s26196091/s1, Script S1, the system-level Monte Carlo in Section 5.7 (system_level_mc.py); Data S1, its per-die results, summary statistics, and run log.

## Abbreviations

The following abbreviations are used in this manuscript:

BBFD	bang-bang frequency/phase detector
CC	calibration chain
CML	current-mode logic
DAC	digital-to-analog converter
DNL	differential nonlinearity
DSM	delta-sigma modulator
DTC	digital-to-time converter
ESD	electrostatic discharge
FD	fine delay
FDSOI	fully depleted silicon-on-insulator
FOD	fractional output divider
INL	integral nonlinearity
LDO	low-dropout regulator
LMS	least-mean-square
LSB	least significant bit
LUT	look-up table
LVDS	low-voltage differential signaling
MC	main chain
MMD	multi-modulus divider
MSB	most significant bit
PDK	process design kit
PLL	phase-locked loop
SAR	successive approximation register
SNDR	signal-to-noise-and-distortion ratio

## Appendix A. Design Parameters

Table A1 collects the design parameters of every block, so that the full set is reported in one place.

**Table A1:** Design parameters, GF 22FDX, $V_{\mathrm{DD}}=0.8$ V.

Block	Parameter	Value
Timing	input clock, LSBs	$f_{\mathrm{in}}=10$ GHz; $T_{\mathrm{LSB}}=24.4$ fs; $T_{\mathrm{CAL}}=12.2$ fs; N≥10
Delay cell (Figure 3)	timing capacitance $C_{\mathrm{tot}}$	2 pF at the timing node: main timing capacitor $C_{\mathrm{cap}}=1.6$ pF + varactor-dilution capacitor $C_{0}=150$ fF + threshold-inverter varactor (165–254 fF)
	discharge current, ramp slope	$I_{\mathrm{dis}}=8$ mA; 4 V/ns
	full-scale start-voltage swing	$V_{\mathrm{ref}}=2^{B}G\;I_{\mathrm{dis}}/C=0.4$ V
	precharge PMOS	W=5μm, L=20 nm, m=2
	M1 cascode / M2 current source	M1: W=5μm per finger, nf 8, m=5 (40 μm total); M2: W=4.875μm per finger, nf 8, m=1 (39 μm); L=20 nm
	M3 gain-boost amplifier / M4 load	M3: W=4.75μm per finger, nf 8, m=4 (38 μm); M4: W=5μm per finger, nf 8 (40 μm); L=20 nm
	M5–M7 bias mirror	M5: W=4μm (1:4 mirror ratio); M6, M7: W=3.125μm per finger, nf 8 (25 μm); L=20 nm
	threshold inverter	trip point $V_{\mathrm{TH}}=350$ mV; PFET (pfet_rf): W=0.75μm per finger, nf 8, m=3 (6 μm); NFET (nfet_rf): W=0.814μm per finger, nf 8, m=2 (6.512 μm); L=20 nm; input-referred noise <10μV
R-2R DAC	resolution; unit resistor	12 bits; R=50Ω, opnpc poly resistor, unit 5μm × 360 nm (32× the initial unit area)
	bit switches	bootstrapped, W=0.56μm per finger, nf 50 (28 μm), L=20 nm; $R_{\mathrm{on}}\leq R/10=5\;\Omega$
	settling	$\tau =R\;C_{\mathrm{tot}}=100$ ps; 9τ=900 ps $\Rightarrow \left(N-1\right)T_{\mathrm{in}}\geq 900$ ps, i.e. N≥10
Fine-delay vernier	construction; code	binary-weighted discharge width; 8 bits, $T_{\mathrm{CAL}}$ step, ±1.56 ps span; $W_{\mathrm{unit}}=5\;\mu$m (nf 1, *m* 1)
Gain bank $C_{\mathrm{tun}}$	capacitor sections	coarse and fine sections, 3 bits each, 3 fF unit: 64 steps, ±4.8% of $C_{\mathrm{tot}}$, one step =150 fs of full-scale delay
	trim section	8 bits, 0.2 fF unit (51 fF range): $\Delta G/G=1.0\times 10^{-4}$ per step, inside the requirement of Equation (16)
	coarse process range	beyond the ±4.8% the capacitor sections span, taken up by the discharge-current setting (Figure 1)
Correction element	code; step	8 bits at 6.1 fs (quarter DTC LSB), the range and granularity the Monte Carlo in Section 5.7 requires
Calibration chain	construction	replica of the main chain, delay element 64× initial area
Digital	SAR; counter; look-up table	8-bit asynchronous SAR; 15-bit comparison counter ($M_{\max}=108\cdot 4^{4}=27,648$); 32× 8-bit table
